# γ-Secretase Studied by Atomistic Molecular Dynamics Simulations: Global Dynamics, Enzyme Activation, Water Distribution and Lipid Binding

**DOI:** 10.3389/fchem.2018.00640

**Published:** 2019-01-04

**Authors:** Manuel Hitzenberger, Martin Zacharias

**Affiliations:** Physics Department T38, Technical University of Munich, Garching, Germany

**Keywords:** γ-secretase, familial Alzheimer's disease (fAD), Molecular dynamics (MD), presenilin, nicastrin, amyloid, intramembrane aspartyl proteases, intramembrane proteolysis

## Abstract

γ-secretase, an intramembrane-cleaving aspartyl protease is involved in the cleavage of a large number of intramembrane proteins. The most prominent substrate is the amyloid precursor protein, whose proteolytic processing leads to the production of different amyloid Aβ peptides. These peptides are known to form toxic aggregates and may play a key role in Alzheimer's disease (AD). Recently, the three-dimensional structure of γ-secretase has been determined via Cryo-EM, elucidating the spatial geometry of this enzyme complex in different functional states. We have used molecular dynamics (MD) simulations to study the global dynamics and conformational transitions of γ-secretase, as well as the water and lipid distributions in and around the transmembrane domains in atomic detail. Simulations were performed on the full enzyme complex and on the membrane embedded parts alone. The simulations revealed global motions compatible with the experimental enzyme structures and indicated little dependence of the dynamics of the transmembrane domains on the soluble extracellular subunits. During the simulation on the membrane spanning part a transition between an inactive conformation (with catalytic residues far apart) toward a putatively active form (with catalytic residues in close proximity) has been observed. This conformational change is associated with a distinct rearrangement of transmembrane helices, a global compaction of the catalytically active presenilin subunit a change in the water structure near the active site and a rigidification of the protein fold. The observed conformational rearrangement allows the interpretation of the effect of several mutations on the activity of γ-secretase. A number of long-lived lipid binding sites could be identified on the membrane spanning surface of γ-secretase which may coincide with association regions of hydrophobic membrane helices to form putative substrate binding exosites.

## Introduction

The protein complex γ-secretase (g-sec) is the only known intramembrane protease requiring an elaborate interplay between four different proteins: nicastrin (NIC), presenilin (PS), anterior pharynx-defective-1 (APH-1) and presenilin enhancer-2 (PEN-2) (Bai et al., 2015a,b; Langosch et al., 2015; Langosch and Steiner, 2017), rendering it the structurally most complex member of this functional family and due to its proposed role in Alzheimer's disease (AD) also the most studied one (De Strooper et al., 2012; Fukumori and Steiner, 2016). It has been established that g-sec is able to process a large number of substrates (Beel and Sanders, 2008; Haapasalo and Kovacs, 2011; Langosch et al., 2015) (as of today more than 90 potential substrate molecules are known Langosch et al., 2015) indicating that one role of this protein complex is the removal of partially degraded proteins from the membrane, thereby preventing their accumulation.

The most thoroughly investigated (Langosch et al., 2015; Langosch and Steiner, 2017) target of g-sec mediated cleavage is C99, containing a single-span transmembrane alpha-helix. C99 is the C-terminal fragment of the amyloid precursor protein (APP) and results from the removal of large parts of the APP ectodomain (Zhang et al., 2011). This preprocessing step, in the case of APP mediated by β-secretase (Vassar et al., 1999), is necessary for sterical reasons: Proteins possessing large soluble extracellular domains are unable to get into close contact with the active site of g-sec (Bai et al., 2015b; Langosch et al., 2015; Langosch and Steiner, 2017). The biological role of APP is mostly in the dark (Deyts et al., 2016) but it is well established that sequential C99 processing results in an intracellular peptide (AICD), several short (mostly three amino acid long) peptides and the Aβ40/42/43/46 fragments (Bolduc et al., 2016).

In patients not suffering from familial Alzheimer's disease (FAD), Aβ40 peptides are the main product of C99 cleavage while the longer variants are yielded in much lower quantities (Zhang et al., 2011). This balance, however, seems to be rather delicate and can be shifted toward the production of longer amino acid chains (predominantly Aβ42) by several factors, such as mutations and changes of bilayer composition or temperature (Holmes et al., 2012; Szaruga et al., 2017). The Aβ>40 fragments are known to be more prone to aggregation than the shorter variants and thus have been found to be the main components of amyloid deposits in the brains of AD patients (Hardy and Higgins, 1992; De Strooper et al., 2012; Langosch et al., 2015; Langosch and Steiner, 2017). Over 200 pathogenic Alzheimer's disease related PS mutations have been reported on www.alzforum.org (affecting 135 different amino acids), as well as over 20 that are situated on C99. Another well studied substrate for g-sec is the Notch ligand/receptor complex, which upon cleavage releases an intracellular fragment leading to the expression of various genes. Aberrant activation of this Notch signaling pathway has been found to promote tumor cell proliferation and is linked to several types of cancer (Rao et al., 2009; Krop et al., 2012).

Structurally, g-sec adopts an N_*in*_ topology, forming a complex with 1:1:1:1 stoichiometry (Bai et al., 2015a,b; Langosch et al., 2015). Because there are two different PS and APH-1 genes - PS-1, PS-2, APH-1a, and APH1b, four different complexes can be formed. PS-1 and APH-1a, however, are the prevalent variants of the respective proteins (Bai et al., 2015b), resulting in NIC:PS-1:APH-1a:PEN-2 being the most common g-sec complex.

**Figure 1:**
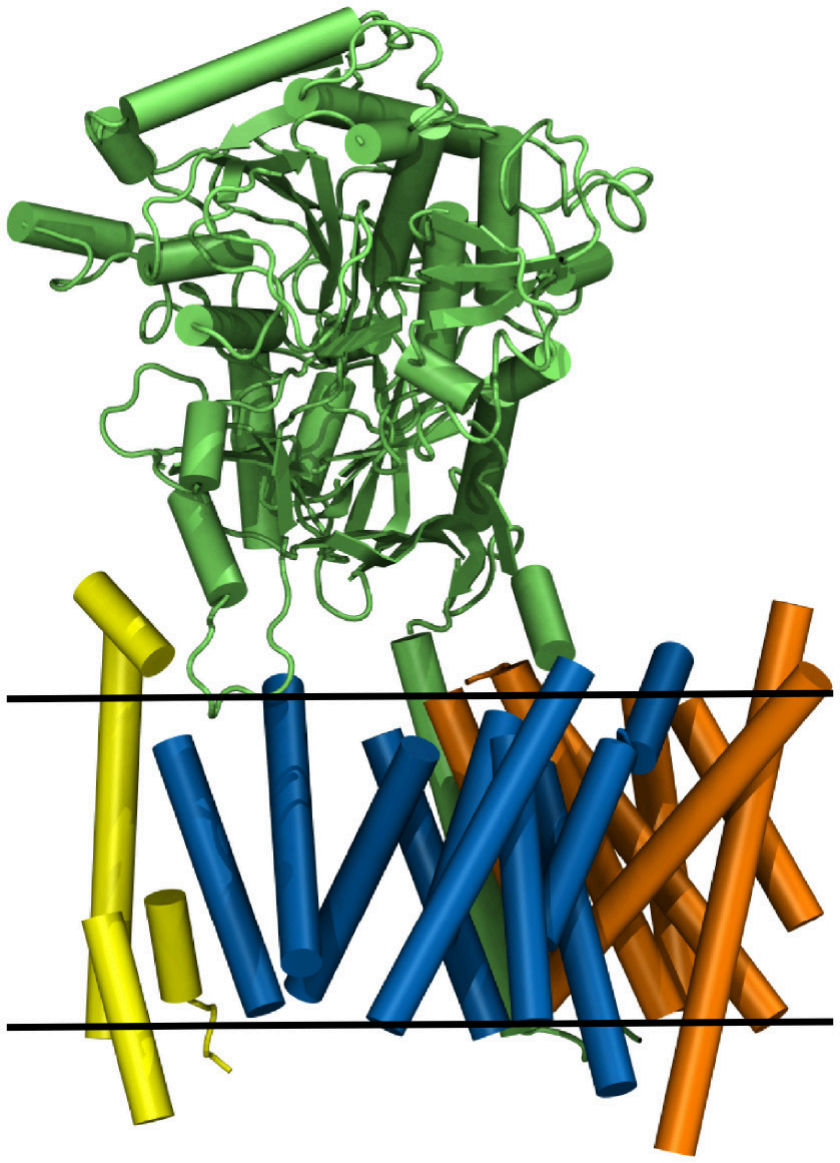
The g-sec complex. Nicastrin (green), presenilin (blue), anterior pharynx-defective-1 (orange), and presenilin enhancer-2 (yellow). The approximate location of the lipid bilayer is indicated by two black lines.

Nicastrin (green structure in Figure 1), a 709 amino acid (AA) long protein, consisting of one transmembrane helix and a large globular extracellular ectodomain (ECD) is believed to play the role of gatekeeper for g-sec, blocking the access to PS for potential substrates with its bulky soluble domain (Bai et al., 2015a; Langosch et al., 2015; Langosch and Steiner, 2017). Presenilin-1 (blue protein in Figure 1), a 467 AA long aspartyl protease, contains the two catalytically active residues D257 and D385 (Wolfe et al., 1999). APH-1a (orange in Figure 1) consists of 265 residues, functions as a scaffolding protein and binds the transmembrane domain (TMD) of nicastrin (Lee et al., 2004). PEN-2 (yellow in Figure 1) has been shown to play a role in the autoproteolytic cleavage of the long cytosolic loop between the transmembrane domains 6 and 7, taking place upon maturation of the g-sec complex (Bai et al., 2015b). With only 101 AA (Francis et al., 2002) it is also the smallest of the four proteins. Even though a lot of insight into the structural and biochemical properties of g-sec has been gained in the last couple of years (De Strooper et al., 2012; Bai et al., 2015a,b; Holmes et al., 2012; Kong et al., 2015; Langosch et al., 2015; Fukumori and Steiner, 2016; Somavarapu and Kepp, 2016; Xu et al., 2016; Langosch and Steiner, 2017; Szaruga et al., 2017), many questions still remain unanswered:

Most soluble proteases cleave their substrates on a (sub)second timescale, whereas g-sec operates much less efficiently, taking minutes to process a single molecule (Kamp et al., 2015; Langosch et al., 2015). Since the chemical process of bond hydrolysis itself takes less than a second in most soluble proteases (Grossman et al., 2011; Langosch et al., 2015), the low turnover rate of g-sec must be the result of a slow process that is necessary to initiate the actual substrate cleavage. The nature of this process is unknown and could involve substrate positioning, conformational rearrangements, frequent enzyme/ligand dissociations or any combination of the aforementioned. Another interesting question associated with the hydrolysis is raised by the fact that the active site of PS is situated deeply inside the membrane region, usually considered to be highly hydrophobic—a property presumably obstructive to a process involving water molecules.

The mechanism of substrate recognition and discrimination is unknown as well. As of now, no structural or dynamical profile attributable to all known g-sec substrates could be identified (Langosch et al., 2015; Langosch and Steiner, 2017). Since g-sec seems to play a major role in familial Alzheimer's disease (FAD), influencing its behavior and thereby forcing it into an Aβ production line where only the more benign 40AA long variant is produced looks to be a very promising endeavor. While complete g-sec inhibition has been shown to have a detrimental effect on health (De Strooper, 2014), preliminary g-sec modulation studies have shown some promise (De Strooper and Chávez Gutiérrez, 2015). Gaining further insight into the exact physico-chemical processes steering C99 processing will aid rational, structure-based approaches to drug design as well as the development of novel gene-therapeutic strategies.

A milestone in the investigation of g-sec was achieved by Bai et al. reporting three-dimensional structures of the complete γ-secretase complex (Bai et al., 2015a,b). These Cryo-EM experiments also uncovered that g-sec exists in a remarkable conformational diversity: Three distinctively different conformational states of the complex have been discovered. They are mainly differing in the distance of the TMDs 6 and 7—bearing the catalytically active side chains and the relative positioning of PEN-2 to PS-1 (Bai et al., 2015b).

Based on the Cryo-EM structures an anisotropic network model (ANM) has recently been constructed to analyze sterically possible large scale motions of the g-sec complex. These studies identified several hinge sites in g-sec and suggested large scale motions of the nicastrin domain relative to the PS-1 that could be involved in positioning of the substrate and promoting cleavage (Lee et al., 2017). Such large scale domain motions were also found in a combination of atomistic and coarse-grained (CG) Molecular Dynamics (MD) simulations of g-sec structures (Aguayo-Ortiz et al., 2017). In addition, the CG simulations suggested transitions between an inactive conformation with a large distance between two Asp residues involved in catalysis and states with the two Asp residues in a geometry compatible with a catalytically active arrangement that was not observed in shorter atomistic simulations (Somavarapu and Kepp, 2017). In the current study we conducted long time scale atomistic MD simulations starting from Cryo-EM derived structures to investigate local and global g-sec mobility and how it might be related to function but focusing also on water and lipid distribution surrounding the g-sec complex. In simulations of the membrane spanning part of g-sec the studies indicate a transition from an inactive arrangement (large distance between Asp residues involved in catalysis) to a potentially active form with a close distance of the Asp residues including also transient binding of water molecules in the active site. It also gives insight into the sterically possible motions of the PS1 TM helices mediating the transition. The active site but also other regions between TM helices appear to be accessible to water several Angstroms away from the boundary between membrane and the aqueous phase. Analysis of the lipid mobility around the g-sec complex revealed several stable binding regions that indicate binding regions for hydrophobic substrate helices. Finally, we interpret the simulation results in light of the potential role of some known mutations in γ-secretase that interfere with activity due to a putative influence on transitions toward the active conformation.

## Materials and Methods

Both simulations (henceforth referred to as system 1 and system 2, respectively) that have been conducted for this study were based on the PDB structure 5FN2 (Bai et al., 2015b) since it is the most complete of all available g-sec structures. In 5FN2, not only transmembrane domain 2 (TMD 2) is fully resolved, also a large patch of the loop 2 region connecting TMD 6 to TMD 7 is visible. All other available g-sec PDB structures miss this loop 2 domain spanning from residue 264 to 278. This region is very close to the putative binding site, therefore its structure and dynamics may be important to the functioning of the enzyme. Unfortunately, structural data on the largest part of this intracellular loop 2 region is still missing because it is outside of the membrane and very mobile. Therefore, this 89 amino acid long region (residues 289 to 377) was restored by CHARMM-GUI and cleaved to enable the simulation of the matured complex.

To enable the physically correct positioning of the proteins in the POPC membrane bilayer, the PDB file was submitted to the PPM (Lomize et al., 2012) server. The starting structure and the simulation protocol was then generated by uploading the reoriented structure to the CHARMM-GUI (Jo et al., 2008; Wu et al., 2014) web server. Since aspartyl proteases require one active aspartate to function as a base and the other one as an acid (Singh et al., 2008), D257 has been protonated. Bai et al. (Bai et al., 2015b) have reported a hydrogen bond between E280 and H163, holding the loop region situated underneath the putative substrate binding site, in place. Because mutation of E280 is the most common cause leading to FAD (Lemere et al., 1996), this structural feature has been stabilized by protonating H163 in the simulations. The rest of the titratable side chains have been left in protonation states, assumed to be the predominant ones under physiological pH. The proteins were embedded in a bilayer consisting of 300 POPC molecules and placed in a periodic simulation box containing 0.15M KCl and approx. 33,000 or 55,000 water molecules, respectively. The target temperature was set to 303.15K, using Langevin dynamics (Goga et al., 2012) with a collision frequency of 1ps^−1^ while the system pressure was kept at 1bar by the Berendsen barostat (Berendsen et al., 1984) and a relaxation time of 0.5 ps. By applying the SHAKE algorithm (Ryckaert et al., 1977), the systems could be propagated by 2.0fs every time step. Non-bonded interactions have been calculated explicitly until a distance of 8Å after which long range effects were accounted for by the particle mesh Ewald method (Darden et al., 1993). Prior to simulation, the aqueous layers have been relaxed by geometry optimization. Preceding a 500ns equilibration phase, the simulation boxes were heated to the target temperature while simultaneously applying pressure and slowly lowering/removing the positional restraints placed on the amino acids and lipid molecules. The proteins were described by the AMBER14SB (Maier et al., 2015) force field, whereas for lipid molecules and water the Lipid14 (Dickson et al., 2014) and TIP3P (Mark and Nilsson, 2001) force fields were used. All simulations were performed utilizing the CUDA (Nickolls et al., 2008) version of the pmemd program, provided with the AMBER16 package (Case et al., 2016). Trajectory analysis and calculation of principle components of motion (PCA) were carried out using cpptraj, which is part of the AMBER16 package (Case et al., 2016) and the results were visualized by VMD (Humphrey et al., 1996). The only difference between the simulations is the absence of the nicastrin ectodomain in system 2 (the TMD of nicastrin was included in both simulations). Due to the bulky nature of the NIC ectodomain, its removal permits a large reduction of simulated amino acids and water molecules. Besides an increase in sampling time due to reduced number of atoms, the putative influence of the NIC ECD to g-sec dynamics could be studied as well. System 1 has been sampled for 1 μs while the evaluation trajectory of system 2 has a length of 3.5 μs.

## Results and Discussion

### System 1: Complete γ-Secretase Complex

**Figure 2:**
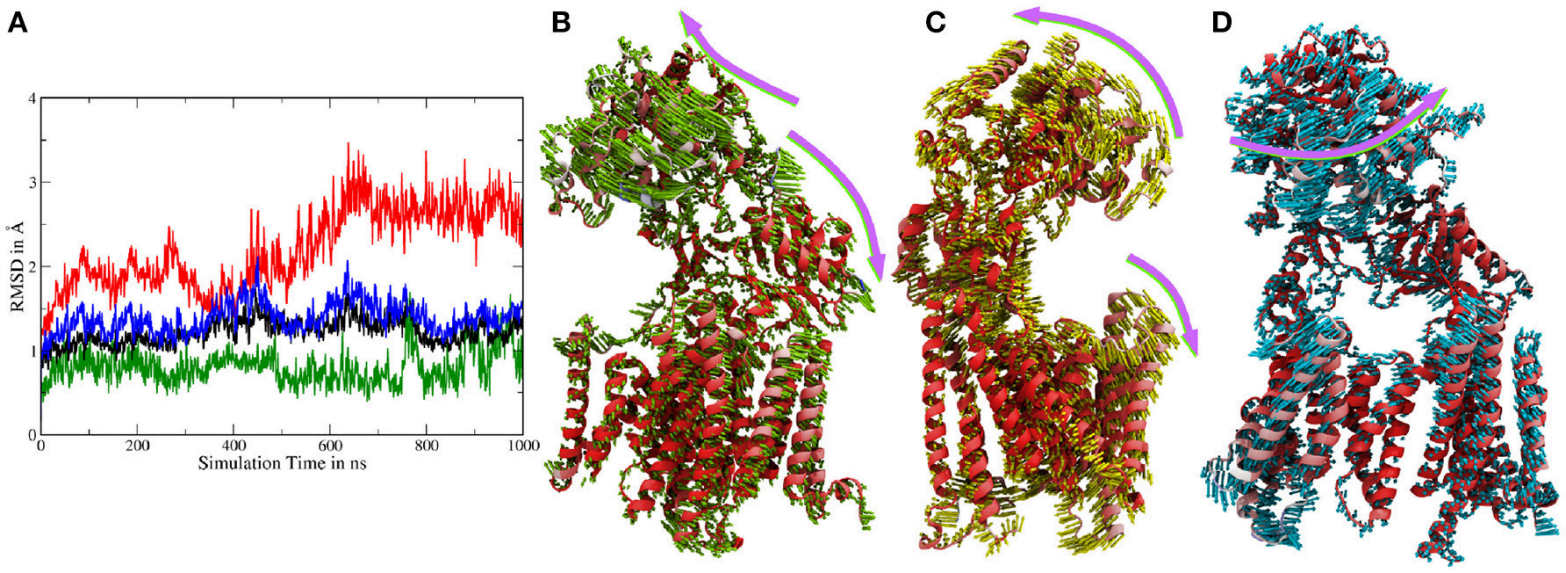
**(A)** Cα-RMSDs calculated for system 1, with the first frame in the trajectory used as reference. Red: RMSD of complete nicastrin; green: TMD of nicastrin; black: all TMDs; blue: PS-1 TMDs. **(B–D)** Principal component analysis of simulation 1 (full g-sec). The mobility of each residue is depicted by the coloring scheme, ranging from red (least movement) via white to blue. The direction and extend of the movement is indicated by small arrows. Due to its high mobility, the intracellular loop 2 was not included in this evaluation in order to emphasize on nicastrin movement. **(B)**: Mode 1. **(C)**: Mode 2. **(D)**: Mode 3.

Atomistic MD simulations were started from the best resolved Cryo-EM structure (pdb entry 5FN2). Root mean square deviations (RMSDs) calculated for the *Cα* atoms of several parts of g-sec compared to the PDB structure indicate that the protein remained close to the experimental structure throughout the simulation (see also Figure S1). During the simulation, the membrane spanning parts fluctuated but on average remained within ~ 2Å of the reference structure (first frame of the sampling trajectory). Since the NIC ECD consists of many mobile loop regions, it naturally exhibits larger deviations than the TMDs situated in the lipid bilayer (which is also more viscous than water). Figure 2A depicts the RMSDs associated with system 1.

In the experimental apo conformation of the PDB-entries 5FN3, 5FN4 and 5FN5 the region beyond residues L262, C263 or P264 of TMD 6 in PS1 (depending on the chosen structure) is not resolved, presumably because of high mobility. However, in the inhibitor bound structure (5FN2) the helix kinks at P264 and goes on for several more residues. According to Bai et al., the presence of the inhibitor rigidifies the structure of g-sec, compared to its apo-form. (Bai et al., 2015b). In the simulation, which was started from 5FN2, an unfolding of the helical region beyond C263 was observed and the chain took on a far more mobile structure in the form of a loop region (Figure S2). This fits very well to the reported apo-structures of g-sec and confirms that the absence of a binding partner near the active site of PS-1 destabilizes this region.

In order to capture coupled global motions occurring during the MD study, principal component analysis (PCA) has been performed on the 1 μs long sampling trajectory of the complete γ-secretase complex. The most pronounced motions are shown in Figures 2B–D. The first principal component revealed that the nicastrin ectodomain consists of two independently moving sub-domains—a small and a large lobe: Most of the TMDs of g-sec and the smaller lobe of the NIC ectodomain are concertedly moving away from the larger globular nicastrin extracellular domain. This behavior indicates potential plasticity of the NIC ectodomain when acting as a binding site for the extracellular terminus of potential substrate proteins (see Figure 2B).

Principal component 2 (Figure 2C) showed that the complex exhibits an opening and closing motion, changing the size of the cavity formed by the ectodomain and the intracellular TMDs. This behavior has previously been reported in coarse grained simulations (Aguayo-Ortiz et al., 2017), elastic network model calculations (Lee et al., 2017) or by experiment (Elad et al., 2015) and may ensure that a broader range of substrate molecules can be processed: The NIC extracellular region has been indicated as a substrate binding site (Fukumori and Steiner, 2016) and in order to play this role it probably has to be close enough to the lipid bilayer to bind and stabilize the membrane-bound substrate. At the same time, however, the cavity has to remain spacious enough to incorporate a sufficiently large part of the substrate's ectodomain—this is necessary to ensure that the intramembrane domain of the substrate can come into close contact with the active site of presenilin. If the cavity formed by nicastrin and the TMDs was of constant size the number of g-sec substrates would very likely be much lower, since fixed NIC-membrane surface distances would allow for only little variety in substrate ectodomain size.

The third largest combined motion of the g-sec complex was a lateral ectodomain movement with respect to the TMDs (see Figure 2D). Upon closer inspection of the mobility of the PS-1 TMD residues in the principal components, it is apparent that TMD2 and the directly connected N-terminal region of TMD3 are very mobile. This finding was not surprising as TMD2 due to its mobility is not visible in many Cryo-EM structures (Bai et al., 2015b), suggesting that TMD2 could function as a possible gate for substrate entry. The video clips of the first three principal components (included as Supplementary Material) depict the relative movement of the different domains and helices of g-sec more clearly.

**Figure 3:**
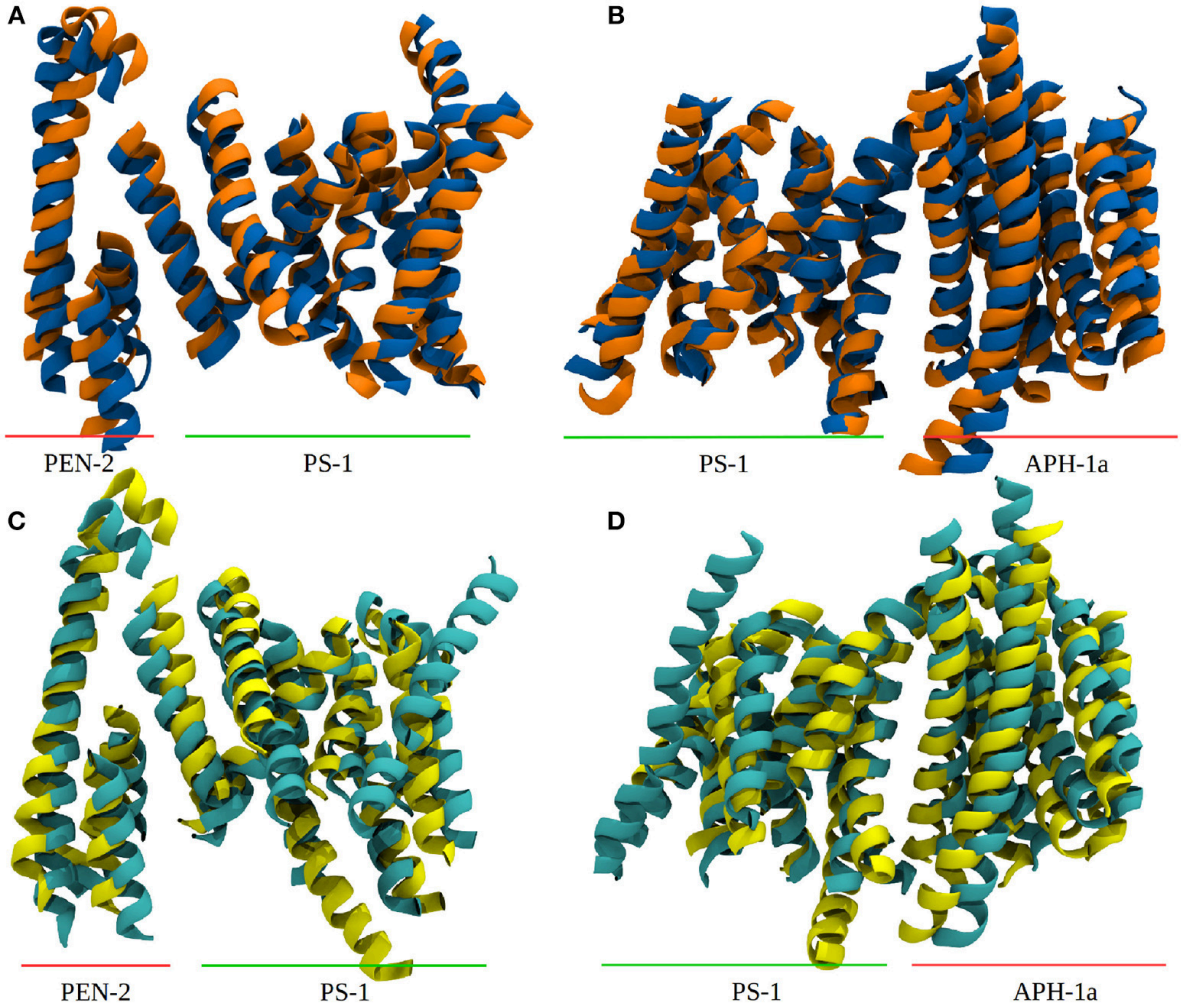
Comparison between system 1 and experimental data. **(A)** Snapshots from system 1, depicting the “in” (blue) and “out” (orange) orientation of Pen-2 by superposition. **(B)** APH-1a tilting with respect to PS-1 in system 1. **(C,D)** Depiction of PDB structures 5FN3 (cyan, class 1) and 5FN5 (yellow, class 3) exhibiting very similar structural diversity. The structures were aligned at the C_α_ atoms of PS-1 TMDs 1, 5, 8, and 9.

The PCA also showed that the conducted simulation reproduced the relative movement of PS-1 and Pen-2, reported by experiments (Bai et al., 2015b) (Bai et al. used the Pen-2 - PS-1 tilting angles in tandem with the active-inactive conformational change to group the reported Cryo-EM structures into three different structural classes). Similar tilting motions with respect to PS-1 can also be reported for Aph-1a. The extend of these motions observed in the simulation has been compared to the PDB structures: The tilt reported in the Cryo-EM structures (5FN2, 5FN3, 5FN4, and 5FN5) has been evaluated by first, aligning them according to the positions of the Cα atoms of PS1 TMDs 4, 5, 7, 8, and 9 and then calculating for each structure, the standard deviation of the angles between helix 3 of PEN-2 and the arbitrarily chosen (yet mutual to all structures) z-axis. In the simulation, the sampled angle changes have been measured by aligning TMDs 4, 5, 7, 8, and 9 in each frame of the trajectory and subsequent calculation of the standard deviation of the PEN-2 - z-axis angle as above. The resulting standard deviations obtained for the PDB files and the simulation were in good agreement with each other (2,47° for the PDB structures and 2.24° for system 1). The deviations of the APH-1a tilting were obtained by the same approach, however, this time the vector used to measure the APH-1a angle with respect to the z-axis was defined by calculating the centers of mass of (i) all the extracellular topside *C*_α_ atoms of the APH-1a TMDs and (ii) of their intracellularly oriented counterparts. This way a mean tilting angle of all the APH-1a TMDs has been determined. The resulting standard deviations of 0.67° (PDB) and 1.71° were in satisfying agreement with each other but suggested that the relative movement between PS-1 and APH-1a was slightly increased in the simulation, compared to the Cryo-EM structures. Plots showing tilting angles vs. simulation time can be found in the supplementary section (Figure S3). Figures 3A,B depict representative snapshots from the simulation, highlighting the sampled protein conformations in the simulated complex that compare well with arrangements observed in the PDB structures 5FN3 and 5FN5 (classes 1 and 3, respectively, shown in Figures 3C,D).

The first three principal components account for 49% of all motions in the simulations. While the fourth component, being a combination of the motions represented by modes 1 and 3, still represented global movement, all other eigenvectors of the covariance matrix referred to very localized loop rearrangements or fluctuations of terminal TMD regions (see Figure S3 indicating the contribution of the first 20 modes).

**Figure 4:**
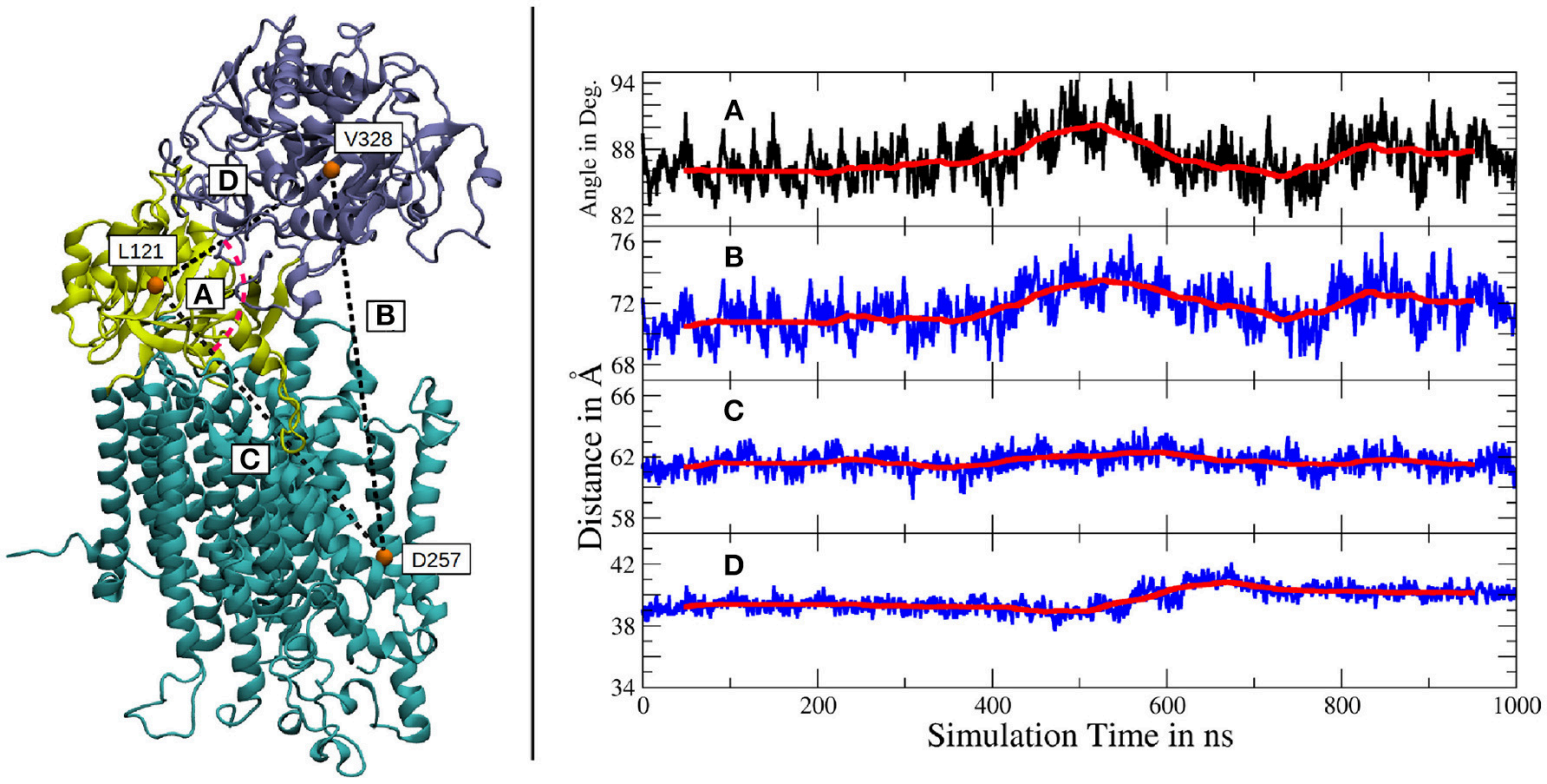
Left panel: Location of the amino acids used for subdomain distance measurements. The large lobe is colored in iceblue, the small lobe in yellow and the intramembrane domains of g-sec are depicted in cyan. Right panel: **(A)** Angle between V328, L121 and D257. **(B)** Distance between V328, situated in the large lobe and D257, one of the catalytically active aspartates. **(C)** Distance change between the small lobe (L121) and the PS-1 active site (D257). **(D)** Distance change between the two ectodomain lobes (L121 and V328). The scaling is the same for all distance plots and a running average (100 points averaging window) has been added for greater clarity (red lines).

The independent motions of the two nicastrin ectodomain lobes relative to PS-1 was further confirmed by a measurement of the distances and angles between amino acids, located either in the large lobe, the small lobe or the active site of PS-1 (see Figure 4). The distance between the large lobe (V328) and the active site of PS-1 (D257) showed two different types of variations: smaller, short lived fluctuations and a much slower but more distinct global movement (indicated by the running average in Figure 4B). The mean extend of this distance change witnessed during the simulation lies in the region of 3 to 4Å. It agrees quite well with the distance variation observed in the high-resolution Cryo-EM structures of Bai et al. (2015b), featuring V328-D257 distance deviations of the same magnitude. However, the larger scale changes reported by Elad et al. (2015) (up to 5 nm) have not been observed in our simulation, it is possible that larger scale global motions may require longer simulations beyond the scale of the present study. The angle defined by V328, L121 (in the small lobe) and D257 changes at exactly the same time as the fluctuations between V328 and D257 take place, clearly showing that this relative large lobe - PS-1 movement is decoupled from the movement of the smaller extracellular subdomain.

The distance changes between the small lobe and the PS-1 active site on the other hand, were of severely reduced extend, only exhibiting relatively low fluctuations and virtually no slow relative movement (Figure 4C). Another indication of decoupled subdomain dynamics of the ectomonain lobes is represented by Figure 4D, where a variation in L88-V295 distance is visible, indicating a conformational change in the nicastrin ectodomain.

It has been reported (Bai et al., 2015b; Aguayo-Ortiz et al., 2017) that PS-1 can adopt two distinct states: One, where the catalytically active side chains are relatively close to each other and a state, characterized by larger D257-D385 distances. For an aspartyl protease to cleave a peptide, besides the presence of the substrate, a water molecule at the right position, as well as two aspartates in a certain (proximal) geometry are required (Singh et al., 2008). During the sampling phase of system 1, PS-1 occupied the active state with a very low distance between both active site aspartates (approx. 8Å). This is surely aided by the fact that D257 was protonated, enabling the formation of a hydrogen bond between both side chains which remains stable for the duration of the simulation. For completion it should be noted that during the equilibration phase, also a different conformation was adopted, where D257 and D385 were separated by a larger margin (= inactive conformation).

### System 2: γ-Secretase Without Ectodomain

**Figure 5:**
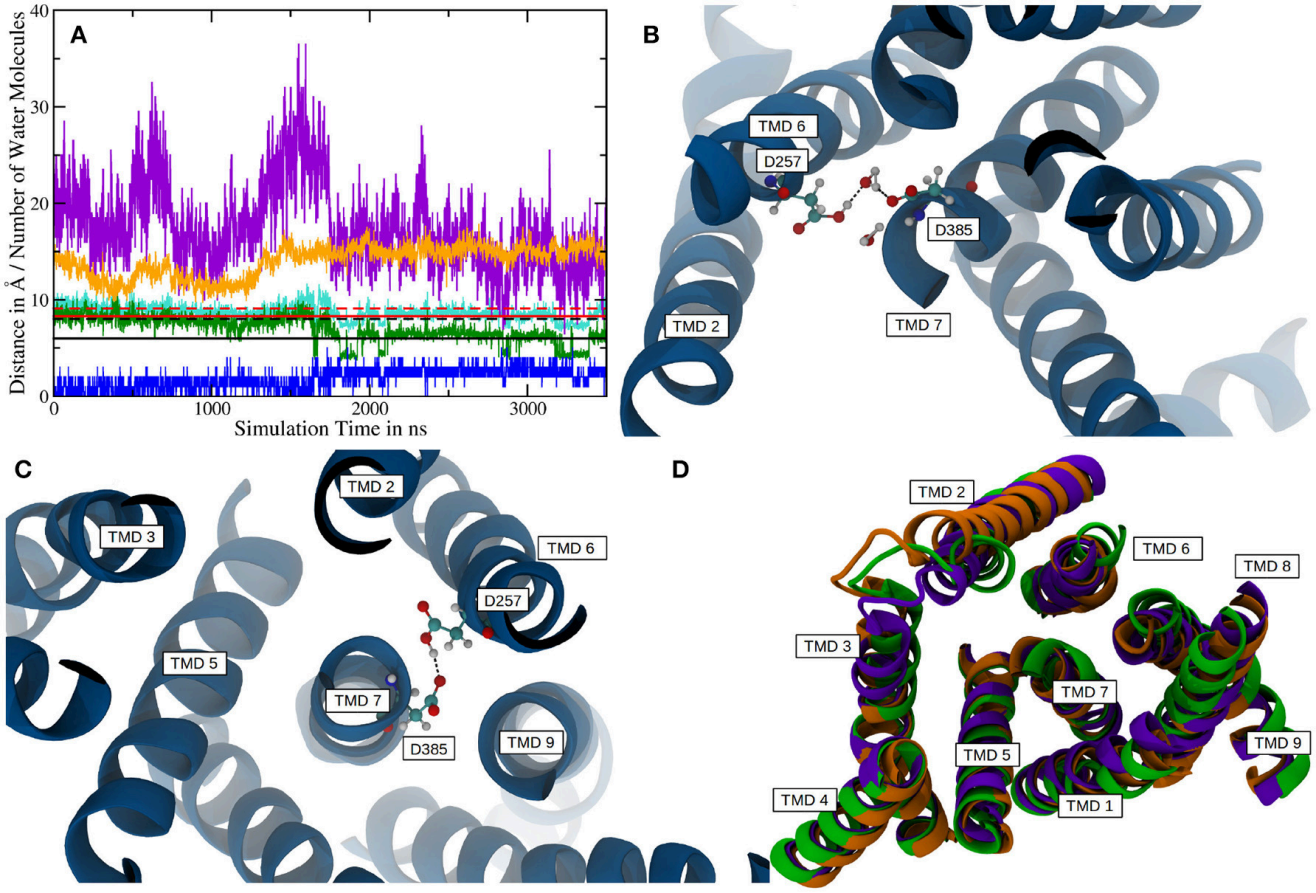
**(A)** Plot depicting distances between certain residues and the number of water molecules within PS-1 in system 2. The Cα distance between D257 and D385 is shown in cyan with the mean distances for the active (solid line) and inactive (dashed line) given in red. The green plot represents the sampled distances between the Cγ atoms of the catalytic aspartates and the black lines indicate the mean separation in the inactive (dashed) and active (solid) states. The distance between V142 (TMD 2) and S169 (TMD 3) is depicted in orange. The blue line is the number of water molecules within 4Å of D257 and D385 and the purple plot indicates the number of water molecules inside the putative substrate binding area of PS-1. Please note, that the purple graph has been scaled down by a factor of two in order to increase comparability. **(B)** The active site aspartates are bridged via a water molecule at Cα distances of 8.6 ± 0.4Å. **(C)** At a Cα separation of 7.5 ± 0.3Å the two catalytic side chains are forming a hydrogen bond. **(D)** Superposition of the mean structures of inactive PS-1 (system 2, green), active PS-1 (system 2, purple) and system 1 (orange). View from the intracellular side.

In order to improve the sampling of the simulation and to investigate the influence of the ectodomain to γ-secretase dynamics, a 3.5 μs (+500 ns equilibration) long simulation of g-sec without the NIC ECD was conducted. The starting structure was taken from a snapshot of system 1 during equilibration. The NIC ECD was removed and the TMDs were embedded in a lipid bilayer consisting of 300 POPC molecules. During the 3.5 μs sampling phase, PS-1 changed its conformation from inactive to active at around the 1.75 μs mark. The mean active-site aspartate (D385 and D257) separations in the inactive state were found to be 9.1 ± 0.6Å (Cα-Cα, with distances up to 11.6Å) and 8.0 ± 0.7Å (Cγ-Cγ). Plots are shown on Figure 5A. The active conformation, on the other hand, was characterized by average Cα-Cα and Cγ-Cγ distances of 8.3 ± 0.6Å and 6.0 ± 1.0Å, respectively (see also Figure 5A, where the red and black solid lines denote the mean aspartate distances in active state, while the dashed lines represent mean values for the inactive conformation). Simultaneously with the putative activation of the enzyme, the separation between the TMDs 2 and 3 increased, indicating a conformational change affecting more than one PS-1 TMD.

**Figure 6:**
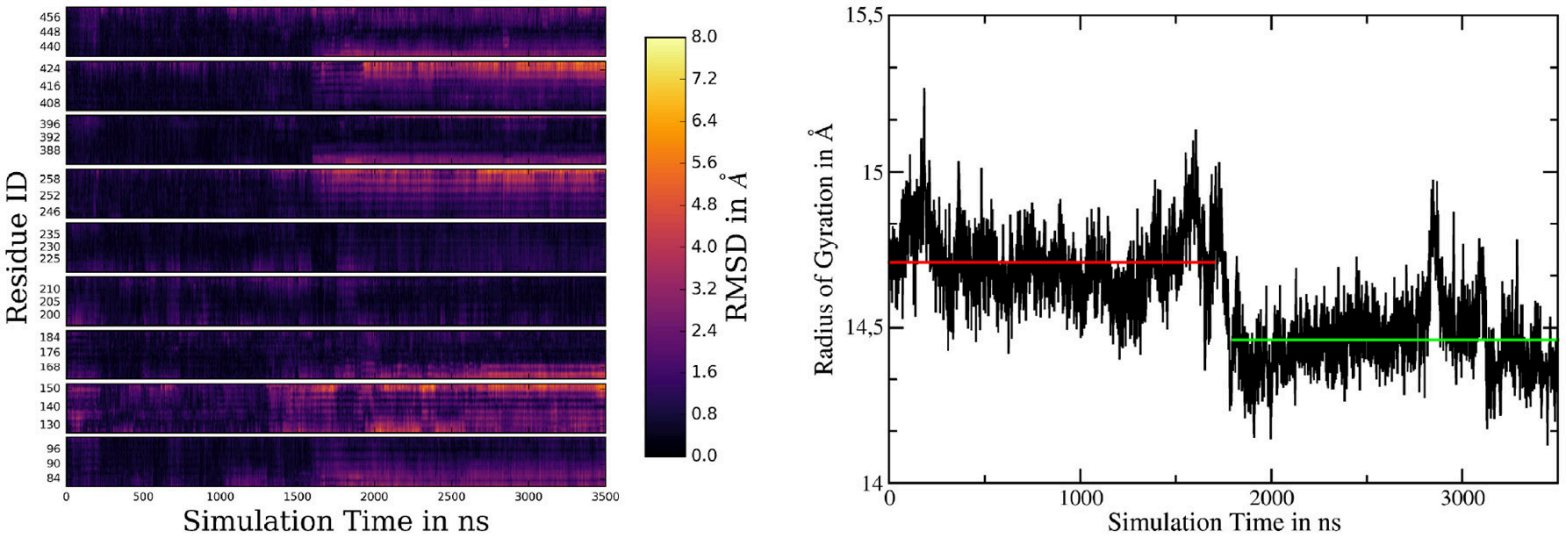
**(Left panel)** Heatmap of per-residue RMSDs of the simulation of system 2, comparing the mean structure of the first 1,000 ns (=inactive conformation) to every frame of the trajectory. **(Right panel)** Radius of gyration of system 2 calculated by considering the Cα atoms of TMDs 1, 6, 7, 8, and 9. The red and green lines denote the mean radii of the inactive and active conformations, respectively.

If one takes a closer look at the catalytic aspartates, it becomes apparent that in active state they can either form a direct hydrogen bond, leading to Cα-Cα distances of 7.5 ± 0.3Å (in 26% of all sampled frames in active conformation) or can be bridged by one water molecule, increasing Cα-Cα separation to 8.6 ± 0.4Å. Snapshots of these respective conformations are shown in Figures 5B,C. The observed D257-D385 separations were in good agreement with the experimental data reported by Bai et al. (finding that active site Cα-Cα distances range from 8.0 to 12.7Å) (Bai et al., 2015b). Another interesting structural aspect can be uncovered by comparing the mean conformation of the first 1,000 ns (inactive state) of simulation 2, the mean conformation of the last 1,000 ns (active state) of system 2 and the mean structure resulting from the 1000ns trajectory obtained for system 1 (active state): The respective structures are shown superimposed on Figure 5D and indicate that transitioning from inactive to active state coincides with the repositioning of TMDs 1, 6, 7, 8, and 9 while TMD 2 and 3 adopt conformational diverse arrangements. The average structures have been generated by aligning the respective trajectories along the heavy atoms of the proteins and subsequently calculating the mean position of each atom in the simulations. This concerted rearrangement of some of the PS-1 TMDs could be further highlighted by calculating RMSDs for every residue in the TMDs in every frame. This was achieved by comparing their positions at each frame to their average position while being in the inactive conformation (the first 1,000 ns of the simulation were taken to calculate the mean inactive structure). The “per-residue RMSDs” depicted on the left hand panel of Figure 6 indicate that the positional shifts of TMDs 1, 3, 6, 7, 8, and 9 coincided with the inactive to active transition of PS-1. From the positions of the Cα atoms of the active aspartates, it seems as if two different principal rearrangements were leading to distances favorable for substrate cleavage: A slight repositioning and rotation of the N-terminal region of TMD 7 (where D385 is located) and TMD 6 (where D257 is located) moving toward the center of PS-1. Transmembrane domain 2 displayed significant mobility throughout the entire simulation that agreed with the experimental observation that the structure of TMD 2 cannot be resolved in several Cryo-EM structures.

Recording the radius of gyration of PS-1 (right-hand panel on Figure 6) demonstrates that the TMD rearrangement upon inactive to active transition was associated with a contraction of PS-1, as the mean radius changed from 14.71Å to 14.46Å.

**Figure 7:**
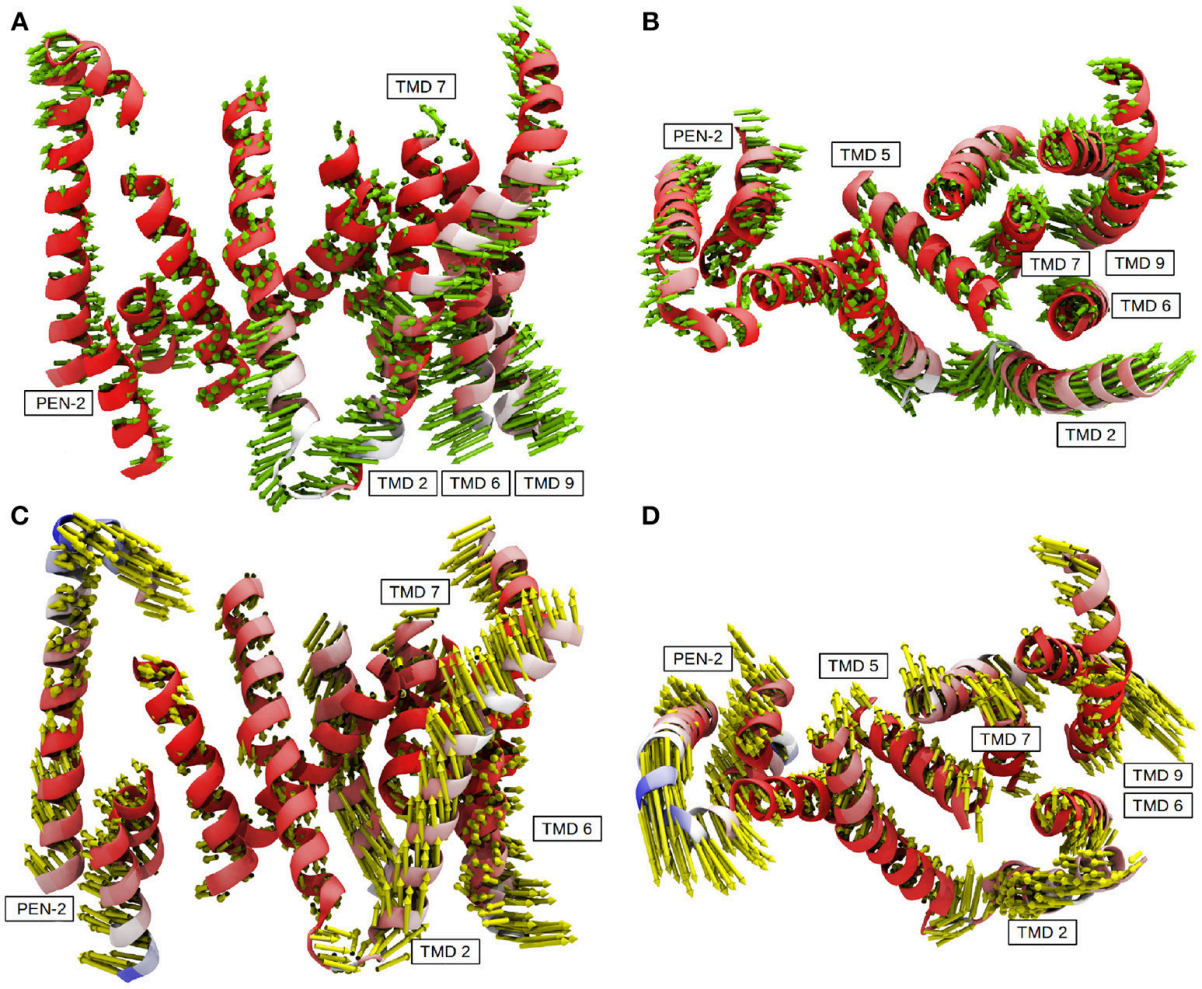
The first two principal components calculated from system 2. **(A)** Mode 1 side view. **(B)** Mode 1 top view (from extracellular side). **(C)** Model 2 side view. **(D)** Mode 2 top view (from extracellular side). Residues colored in red exhibit low mobility while blue indicates strong fluctuations.

The contraction of the intracellular side of PS-1 could also be witnessed by a principal component analysis performed on the 3.5μs sampling trajectory including the TMDs of PS-1 and PEN-2: In mode 1 (Figures 7A,B) TMDs 1, 6, 7, 9 of PS-1 and the PEN-2 α-helices were moving toward each other, thereby reducing the intra-domain distances (and with them also the separation between D257 and D385). PS-1 TMD 8, on the other hand, moved away from the rest of the presenilin helices.

The second most important of the concerted movements is depicted in Figures 7C,D: The distance between the catalytic aspartates remained constant but PS-1 TMDs 4, 5 and to a lesser extend 3 along with PEN-2 moved in different directions than the other PS-1 TMDs. This conformational change was decoupled from the transition between the active and inactive states and confirmed the structural classes reported by Bai et al where PEN-2 tilting also seemed to be independent from the D257-D385 distance (Bai et al., 2015b). Movie clips showing the coupled motions are provided as Supplementary Material. The first two modes of the PCA accounted for 47% of the overall mobility sampled by the evaluation trajectory. The remaining modes, however, emphasized strongly on fluctuations of terminal TMD domains, mainly involving TMD 2 (Figure S4 depicts the contribution of the first 20 modes to the total mobility).

**Figure 8:**
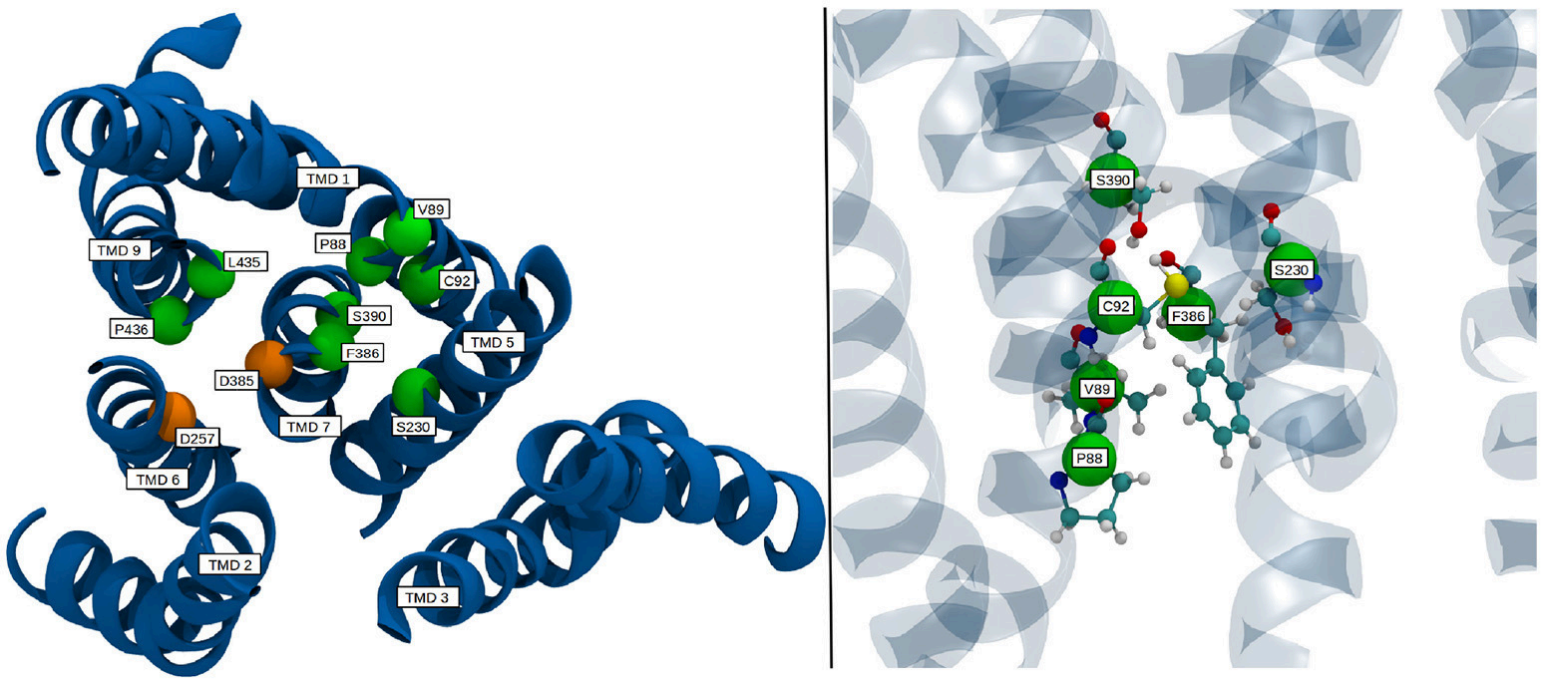
Location of select mutation sites on the TMDs of PS-1 which are known to severely reduce the catalytic activity of γ-secretase. TMDs are colored in blue, mutation sites are depicted as green spheres, while the catalytic residues are shown in orange.

Known pathogenic mutations in PS-1 lead to different results regarding g-sec activity: A change of overall substrate processivity—mainly a decrease, sometimes even to the point of inactivation, or a shift of the ratio of product amyloids (very often increasing Aβ42 levels). Frequently, also a combination of the above effects is witnessed (Sun et al., 2016). Many of the mutations resulting in reduced or abolished g-sec activity are located at the interface of adjacent TMDs. Such mutations can be expected to destabilize presenilin or even prevent correct positioning of the respective TMDs during protein synthesis or folding. It is, however, likely that some of the mutations have a more subtle effect on the PS-1 structure and simply interfere with the relative positioning of the catalytic residues, thereby influencing important conformational changes. One such mutation site is F386, situated on TMD 7, right next to D385. As illustrated in Figure 8, the side chain of this residue is very close to five other known mutation sites (Sun et al., 2016; Szaruga et al., 2017), S390, S230, C92, V89, and P88. While residues P88, V89, C92, and S230 outline the binding pocket of F386, S390 helps to position C92 by acting as a hydrogen bond acceptor. During the simulation, upon activation of the enzyme, F386 “plugged” into the binding cavity, and anchored TMD 7 to TMD 1, thereby impacting the positioning of D385 (see also Figure 6). Changing the sterical properties of this binding site or mutating F386 to a serine can be expected to have an impact on this behavior, hinting at why these mutation sites severely lower the catalytic capabilities of g-sec. Residues P88, V89 and C92 are located on TMD 1 which explains why the positioning of parts of TMD 1 is connected to the inactive-active conformation change of PS-1 (see also right panel on Figure 6). Other residues that may directly influence the positioning of the catalytically active aspartates are L435 and P436. While the mutation of L435 to a phenylalanine can increase the sterical barrier for TMD 7 twisting, changing P436 into a serine might influence the catalytic capabilities of D257 and D385 because S436 would be capable of forming hydrogen bonds with their side chains.

**Figure 9:**
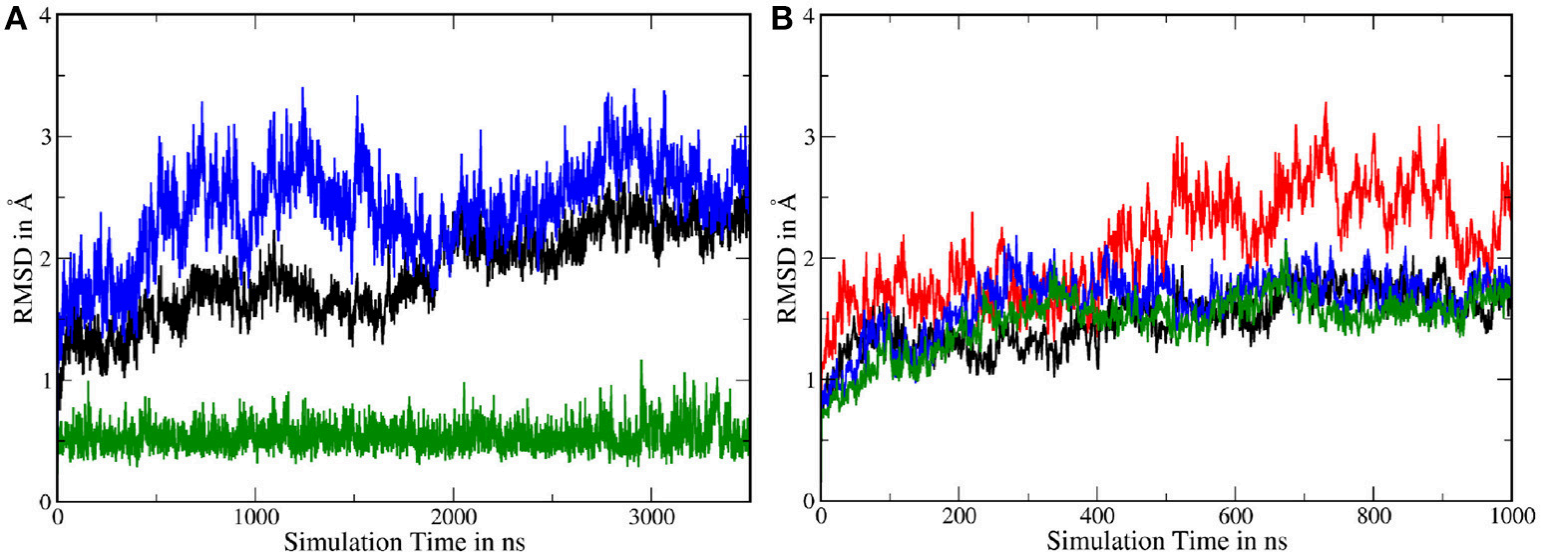
**(A)** Backbone RMSD with respect to the first frame in the trajectory. Green: nicastrin TMD; black: PS-1 TMDs; blue: all TMDs. **(B)** Backbone RMSDs of the split trajectory. Black: PS-1 TMDs (inactive conformation); red: all TMDs (inactive conformation); green: PS-1 TMDs (active conformation); blue: all TMDS (active conformation).

In order to assess the stability of the protein complex and compare it to system 1, several *C*_α_ RMSDs have been calculated. As can be seen in Figure 9A, the fluctuations of the TMDs were considerably higher in system 2, compared to system 1. To isolate the contribution leading to this increased mobility and to improve the comparability to the shorter system 1 simulation, the 3,500 ns long trajectory has been split into two 1,000 ns long trajectories: One trajectory where PS-1 is in inactive state and a second with PS-1 in the (presumably) catalytically active conformation. The transitory part in the middle of the complete trajectory has been left out in order to remove the TMD rearrangements from the dataset (as such a conformational change was not occurring in system 1). The RMSDs resulting from the analysis of these new trajectories showed that the deviations were of the same magnitude as in system 1, with the only exception being the non-PS-1 TMDs in the first 1,000 ns (= inactive state) of the simulation (see Figure 9B), suggesting that the inactive conformation of PS-1 may lead to a slightly less stable protein complex.

**Figure 10:**
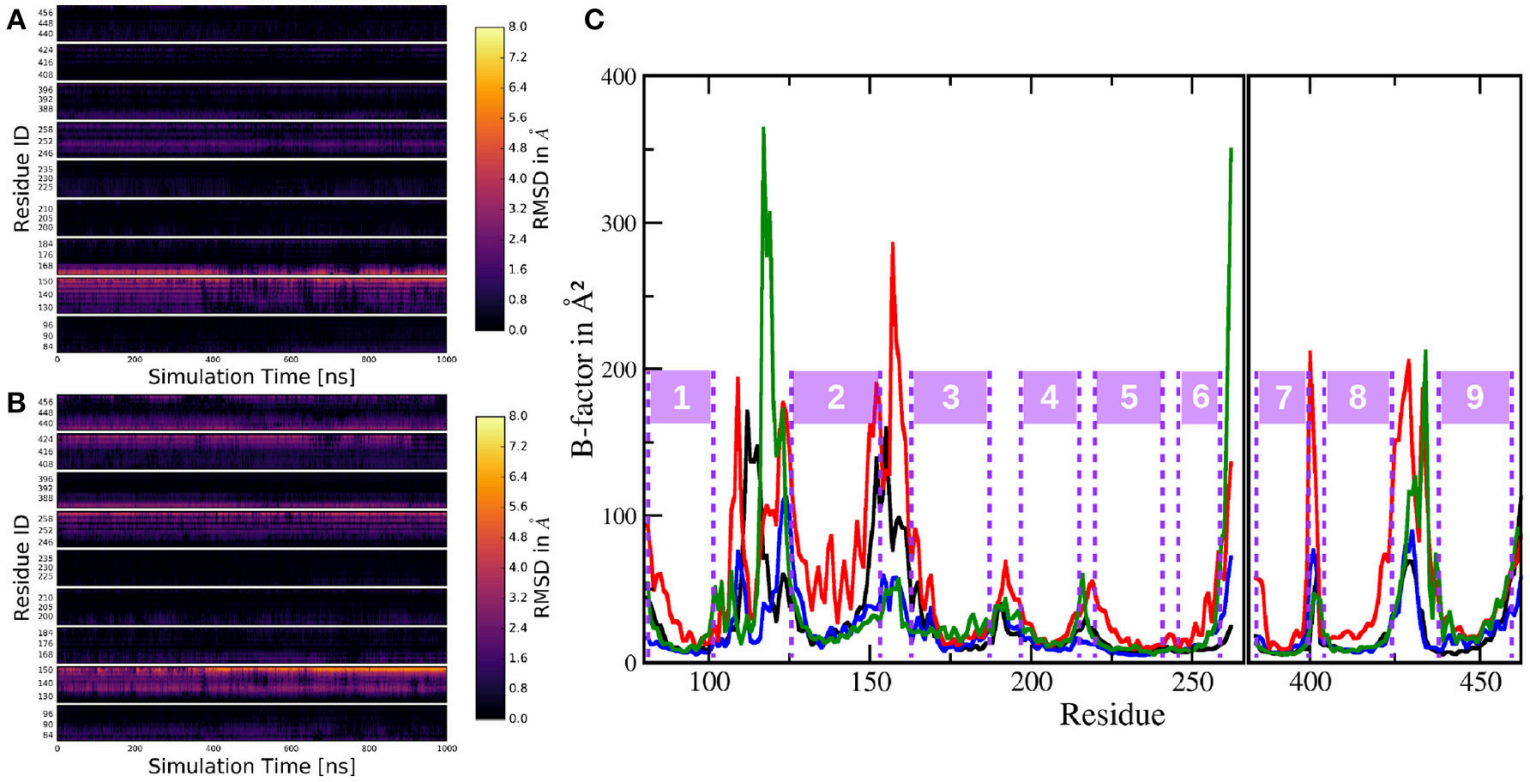
Per-residue RMSD heatmap plots comparing: **(A)** The simulation of system 1 to the mean structure of the active conformation of system 2. **(B)** The trajectory of system 1 to the mean structure of the inactive conformation trajectory of system 2. **(C)** Cα B-factors of the residues in PS-1, excluding the highly mobile loop 2 region. Black: system 1; red: system 2 (complete trajectory); blue: system 2, active conformation (last 1,000 ns of system 2 sim.); green: system 2, inactive conformation (first 1,000 ns of system 2 sim.).

The primary (known) role of the ectodomain of nicastrin is gate keeping the g-sec complex, thereby prohibiting the processing of substrate TMDs before the shedding of their ectodomains. It is also known that nicastrin plays an essential role in stabilizing the g-sec complex (Zhang et al., 2005). To assess the complex stability compared to the simulation of full γ-secretase, PEN-2 and APH-1a tilting motions have been calculated with the same method as for system 1 and with a standard deviation of 2.68° for PEN-2 and 1.68° for APH-1a the results suggest that the absence of the NIC ECD does not destabilize the rest of the protein assembly (values for system 1 are: 2.24° and 1.71°, respectively). Therefore it seems very likely that the stabilizing effect of NIC is due to its transmembrane helix. To further elucidate the role of the ectodomain of nicastrin, the structure and dynamics of PS-1 in systems 1 and 2 have been evaluated and compared to each other. One way of comparing structural features is through per-residue RMSDs, calculated for each frame in the trajectory. The heatmap plot in Figure 10A has been generated by first calculating the RMSDs of all residues in the simulation of system 1 (against the mean structure) in order to establish the "natural" fluctuations of the system (i.e., the noise). In a second step the per-residue RMSDs of simulation 1 were calculated against the mean structure of the last 1,000 ns of simulation 2 (= the active conformation). Subsequently, the per-residue RMSDs of the first step were subtracted from the second data set in order to plot only the (absolute) deviations that occur in addition to the “natural” fluctuations. This plot showed that for the most part, structurally the two simulations were very similar, again with TMD 2 and the N-terminal end of TMD 3 being the exception (and to an extend also TMD 6).

A similar comparison this time, between the inactive conformation of system 2 and simulation 1 resulted in Figure 10B. In this case, the heatmap plot indicated larger deviations between the two instances of PS-1: Additionally to TMD 2 (and 3), also the lower parts (= pointing toward the intracellular region) of TMDs 6, 7, 8, and 9 showed significant aberration throughout the trajectory. This structural evaluation fits nicely to the superposition of the mean structures of trajectories 1, 2 (active) and 2 (inactive) in Figure 5D, indicating the same structural differences. The dynamics of the two systems can be assessed by calculating B-factors for all PS-1 residues (excluding the very mobile loop 2 region). The resulting plots for systems 1 and 2 are shown on Figure 10C.

Evidently, the B-factors of all the TMDs are very low and approximately of the same magnitude in all compared trajectories. The only obvious exception is TMD 2 in the complete trajectory of system 2 where the Cα atoms clearly exhibited heightened mobility. Another difference between the (shorter) simulation including the NIC ECD and the complete trajectory of system 2 was the mobility of the loops connecting the C-terminal TMDs 7, 8, and 9. Upon closer inspection, however, also TMDs 6, 7, 8, and 9 displayed slightly increased dynamics in the complete trajectory of system 2. To put these results into perspective one has to consider the fact that the simulation of system 2 is more than twice as long as the one of system 1 and as has already been discussed, PS-1 undergoes a conformational change including the (relative) rearrangement of several TMDs. Furthermore, TMD 2 is known to be highly mobile, so increased positional fluctuations in this part of the protein are not really surprising. For a more balanced comparison between the shorter and the longer simulation, trajectory 2 has been divided into a 1,000 ns inactive conformation state and a 1,000 ns long active conformation state, while the TMD rearrangements taking place in approx. the middle of the simulation were omitted from the data (these trajectories were exactly the same as those that have been used to generate the additional RMSD plots above). The B-factors corresponding to these new trajectories are shown of Figure 10C as green and blue lines, respectively. These split-trajectories lead to the verdict that the mobility of PS-1 TMDs is not affected by the presence or absence of the NIC ECD: The resulting B-factors of the shorter system 2 trajectories fit nicely to the B-factors calculated for the TMDs in the simulation with complete nicastrin. The case of the loop regions is a bit more complex: The data suggested that (i) the active form of PS-1 exhibits lower fluctuations compared to the inactive conformation, (ii) the loop (and adjacent TMD regions) connecting TMDs 2 and 3 seemed to be of greatly reduced mobility in the simulations without the NIC ECD if inactive-active conformational changes are taken out of the picture. Since, however, the evaluation of the complete simulation 2 trajectory showed greatly increased mobility in this exact region, differences in B-factors concerning the region connecting TMDs 2 and 3 are insufficient to be taken as prove for external influences. These aberrations were very likely just of statistical nature and due to the fact that not all evaluation windows used for the respective B-factor calculations caught the same extent of the configurational changes. Altogether, the available data suggest only a small influence of the ECD of NIC on the structure and dynamics of PS-1.

**Figure 11:**
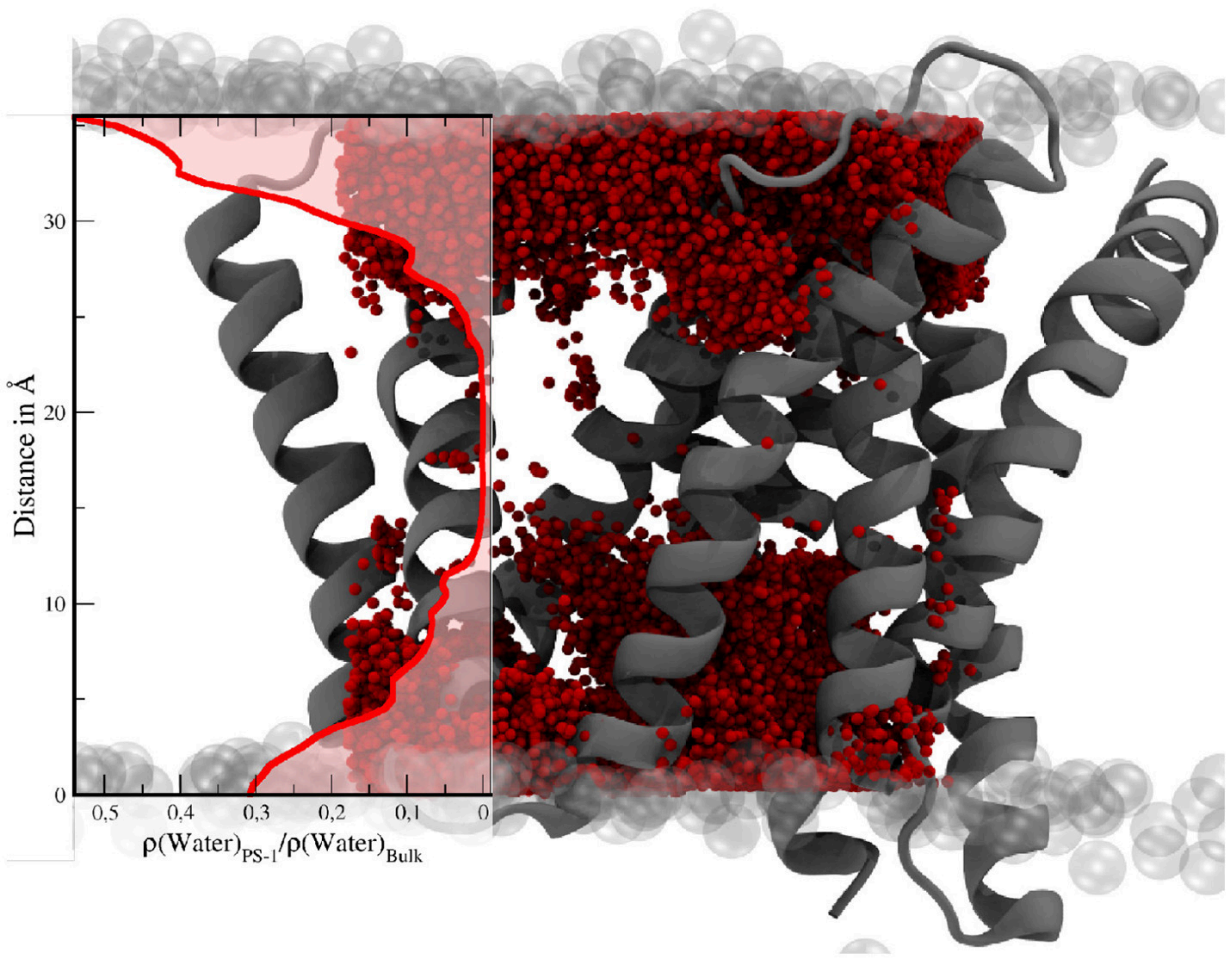
Water molecules within a box of 30 × 28 × 36Å in the TM region of PS-1. The transparent gray spheres indicate the edge of the membrane domain. The red spheres are water molecules. The visualization of the water distribution has been generated by superimposing the positions of all water molecules within the given boundaries at every tenth evaluation frame of the simulation. The graph on the left shows the mean water density (*N*_*water*_/*Volume*) in PS-1, relative to the bulk region.

Apart from conformational transitions, one of the questions in the context of intramembrane proteolysis is the availability of a sufficient number of water molecules, not only to facilitate the hydrolysis but also to stabilize putative transition states and the (putative) unwinding of the alpha helical substrate molecule. In order to investigate its hydration properties PS-1 has been approximated by a box with a volume of approx. 30,200Å^3^. Subsequently, the mean number of water molecules within that cube was calculated by averaging over all 3,500 sampling frames. This evaluation showed that a mean number of 123.2 (with 87 being the lowest count and 167 the highest) water molecules were situated somewhere between the TMDs of PS-1 and with that also inside the membrane. The water distribution, depicted by Figure 11 is not homogeneous and the number of solvent molecules rapidly diminishes in the vertical center of the protein.

**Figure 12:**
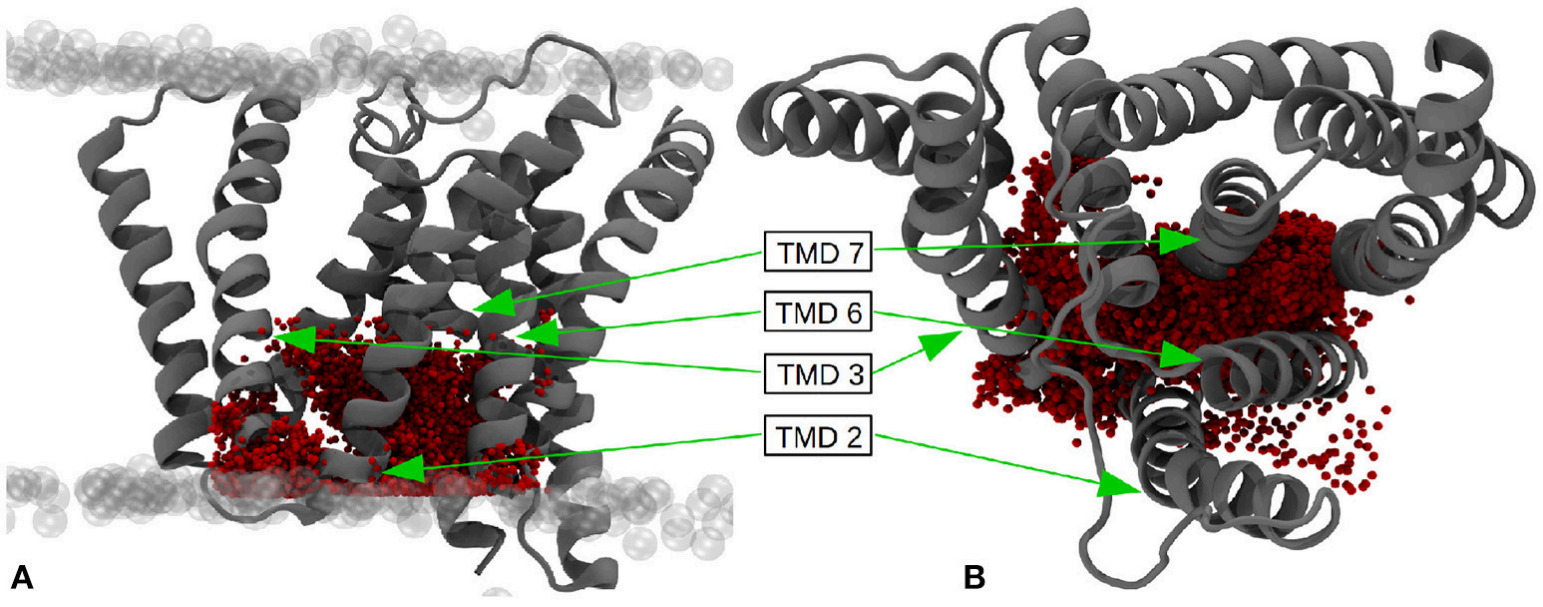
**(A)** Water molecules within the putative binding region of the substrates (27 × 18 × 16Å) - side view. **(B)** Top view of the putative binding region. The pictures have been generated by superimposing the positions of all water molecules within the given boundaries at every tenth evaluation frame of the simulation. The extracellular side is at the top.

Since the active-site aspartates are situated at the intracellular side of the g-sec complex, the water molecules located in this region can be expected to play a larger role in processing and stabilizing the substrate. Therefore, the number of water molecules residing inside the area around the active site—presumably coinciding with the region where the C-terminal part of a substrate molecule is located, has been ascertained as well. The box chosen to represent the putative binding and processing site was approx. 7,800 Å^3^ in size and incorporated a mean value of 34.8 water molecules, ranging from 13 to 73 (see also Figure 12).

Intriguingly, if the number of water molecules is plotted against simulation time, an abrupt change is apparent (purple line in Figure 5A). This change in number of water molecules coincided with the transition from inactive to active state PS-1. Since the box volume remained constant, a change of water accessibility to the binding region must be responsible for this behavior. This finding can be explained by the proposed contraction of the TMDs during the conformational transition of PS-1 to the active form. The plot also highlights that the fluctuation of the number of water molecules close to the cleavage site was distinctly lower in the active conformation, again suggesting a more rigid conformational state (the standard deviation changes from 4.4 to 3.0). The water molecules directly responsible for the hydrolysis of the substrate are of course those that are very close to both catalytic aspartates. As shown by the blue line in Figure 5A, the number of water molecules within 4Å of both D257 and D385, changed from a mean 1.0 (inactive conformation) to 2.6 (active conformation), indicating structural properties more favorable for substrate processing.

One of the open questions regarding g-sec and its processing of single-span TMDs is the location of the substrate binding site(s). In addition to the active site (leading to immediate cleavage), the existence of several exosites responsible for the recognition and recruitment of substrate TMDs has been heavily indicated by a number of experimental studies (Esler al., 2002; Tian et al., 2002; Kornilova et al., 2005; Fukumori and Steiner, 2016). The identification of such sites could help to elucidate the path along which C99 and other substrates enter the active site of PS-1, thereby enabling the analysis of the role played by many of the known PS-1 FAD mutations. Although fatty acid chains of POPC lipids differ quite substantially from TMD α-helices, their main mode of interaction with transmembrane proteins is similar—namely through dispersion effects in the apolar bilayer region and polar interactions at the lipid-water interface. Simple apolar interactions are rather unspecific (especially when compared to hydrogen bonds or salt bridges) and it can be argued that protein regions that are able to immobilize and bind POPC lipids should also be able to form substantial interactions with transmembrane α-helices. Insight into the number and position of such interaction patches was gained by measuring the mobility of all lipids in the simulation box. The ones that showed a very small deviation from their mean position can be regarded as situated at a protein region favorable for binding hydrophobic species (with polar headgroups). In order to achieve this, all subsequent frames of trajectory 2 have been aligned to the first frame with respect to the Cα atoms of the protein TMDs. Subsequently, the heavy-atom B-factors of all fatty acid chains have been calculated.

**Figure 13:**
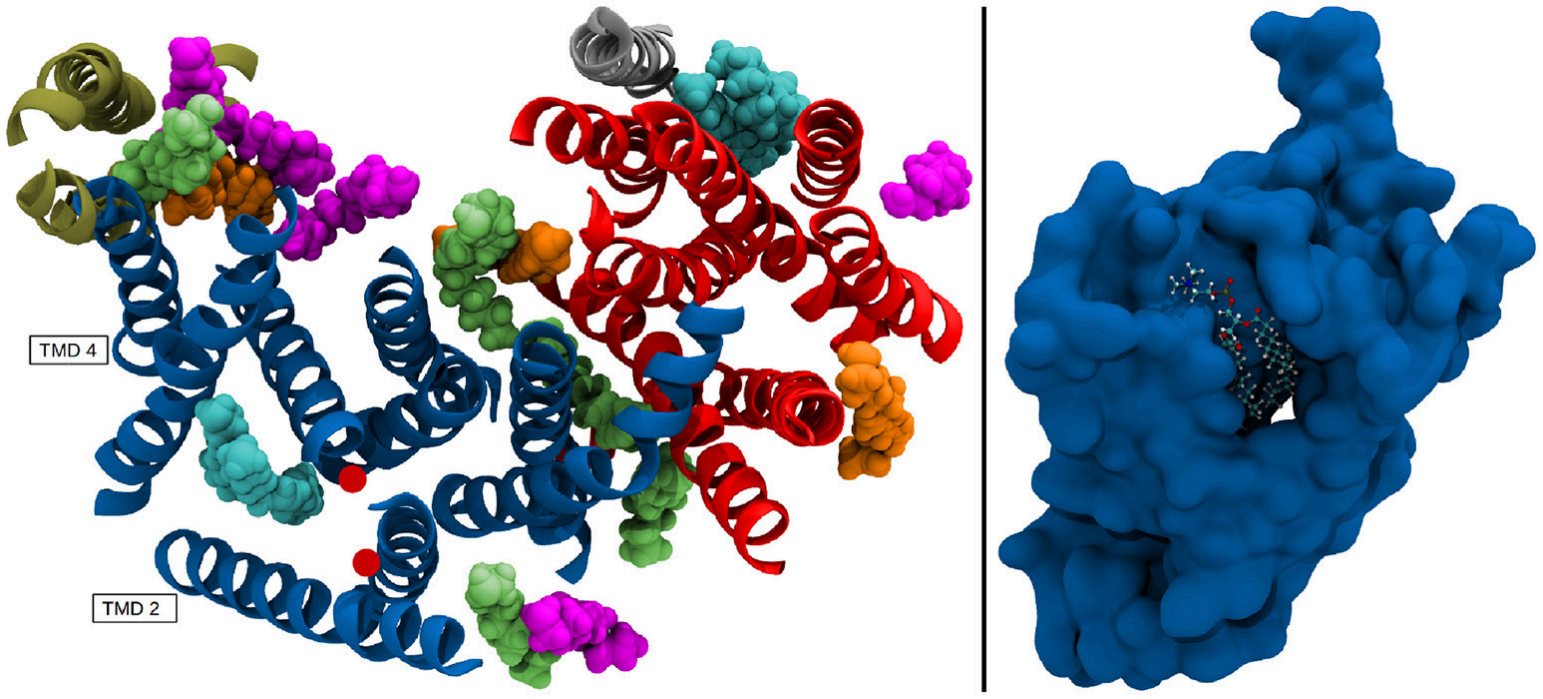
**(Left panel)** TMDs of γ-secretase with strongly immobilized lipids. The TMDs of PS-1 are colored in dark blue, PEN-2 is depicted in beige, APH-1 in red and nicastin in gray. The lipids are colored according to their B-factor: Cyan: 156 to 250Å^2^; orange: 405 to 519Å^2^; green: 610 to 732Å^2^; purple: 785 to 1026Å^2^. The active site residues D257 and D385 are marked by red dots. **(Right panel)** Van der Waals surface depiction of presenilin-1 with the strongly bound POPC molecule in the cavity.

The resulting positional deviations indicated that lipid mobility in the system was very diverse. Generally, most POPC molecules were able to freely travel around the bilayer in the simulation box, however, some lipids were evidently held in place by interactions with one ore more of the g-sec subunits. These lipids have been identified by their low B-factor and the immobilization of their fatty acid chains has been confirmed by visual inspection of the trajectory. The left panel on Figure 13 shows the seventeen lipids that exhibited the lowest B-factors in the simulation. All of the depicted fatty acid chains remained trapped in position and did not diffuse through the bilayer.

The four fatty acids showing the lowest B-factor (157–250Å^2^) depicted in cyan, were situated at two different positions: One lipid (consisting of two fatty acid chains) was bound between the TMDs of nicastrin and APH-1a but since this patch is very far away from the active site it is unlikely that a putative substrate entry path starts from there (let alone it being the active site). The second binding site was intriguingly coinciding with the cavity formed by TMD 2, TMD 3, and TMD 5 in which a co-purified helix had been identified by Bai et al. (2015b). This cavity (see also Figure 13, right panel) remained stable for the duration of all the conducted simulations and is outlined by many of the known mutations reportedly leading to early onset Alzheimer's disease (cf. www.alzforum.org). These circumstances make a strong case for this region being an important substrate binding site, perhaps even the active site.

Another area often suspected to be involved in substrate adoption consists of TMDs 2, 6, and 9 (Tomita and Iwatsubo, 2013; Fukumori and Steiner, 2016). This suggestion is also supported by the present data as two fatty acids chains belonging to the same lipid were binding to that specific region. However, the color scheme (green: B-factor between 405 and 519 Å^2^; purple: B-factor between 785 and 1025 Å^2^ in Figure 13 indicates that the immobilizing effect was lower and one of the two chains was bound with higher affinity than the other. Upon visual inspection of the simulation it became evident that most of the time only one of the two fatty acids was interfacing with the protein, with very few instances where both were able to bind simultaneously. This hints at slightly unfavorable conditions for binding larger entities at this position. Nevertheless, this location is easier to access than the cavity at TMD 2, 3, and 5, as there is a cytosolic loop connecting TMDs 2 and 3, requiring deformation of the substrate helix upon entering. A potential substrate helix bound at either of the two positions would be very close to the active site. Another interesting accumulation of lipids was situated at PEN-2 and PS-1 TMD 4 where a number of immobilized fatty acid could can be found, indicating that this region might play a role as an exosite, capturing potential substrates moving freely in the membrane.

## Conclusions

Two atomistic (1 to 3.5 μs long) simulations of the γ-secretase complex have been conducted. with evaluation trajectories that were up to 3.5 μs in length. To our knowledge these are the longest purely atomistic computer simulations of the complete g-sec complex conducted to date. All simulations exhibited modest positional deviations throughout the sampling phase, indicating that the employed force fields were able to maintain stable conditions and keep the structure of the protein complex close to its native Cryo-EM-deduced structure. The existence of different conformational classes with respect to the distance between the active site aspartates and the relative position of PS-1 and PEN-2, as predicted by experimental means (Bai et al., 2015b), were also observed in the simulations. Additionally, similar tilting motion as exhibited by PEN-2 were also witnessed for APH-1a in agreement with available high resolution Cryo-EM structures.

Evaluation of the dynamical aspects of the complete complex in a POPC bilayer further confirmed the existence of a pronounced “up/down” and “left/right” movement exhibited by the NIC ECD. This sort of relative ecto-TMD movement has previously been suggested by simulations conducted with coarse grained (Aguayo-Ortiz et al., 2017) or elastic network models (Lee et al., 2017) of g-sec.

Since g-sec has been simulated with and without the bulky nicastrin ectodomain it was possible to investigate the influence on the dynamical and structural properties of the catalytic subunit of the complex. On the time scale of the present simulations no significant effect of the ectodomain on the structure and dynamics of presenilin-1 has been observed.

In one of the simulations a conformational change leading from catalytically inactive PS-1 to the active conformation has been sampled and the associated distinct motions characterizing the transition have been identified: A reduction of the distance between TMD 6 and 7 by an inward movement on the part of TMD 6, as well as a rotation of the N-terminus of TMD 7, leading D385 to face D257. Also, the observation of an active conformational arrangement over μs time scale indicated that such a state is at least transiently accessible also in the apo state of the enzyme. RMSD, mobility and water hydration data strongly suggested that the active conformation of g-sec is more rigid than the inactive form. It has recently been speculated that some FAD mutations may lead to a destabilization of the protein fold which in turn could have an impact on its proteolytic capabilities (Szaruga et al., 2017). The conformational change sampled in this study suggests that even the wildtype of γ-secretase exists in conformational states with differing rigidity. Mutations at neuralgic positions can be expected to shift the balance between these states also in the direction of the state with more loosely associated TMDs. This could have an impact on both, the enzyme-substrate complex stability and the length of the interval between two processing steps (because g-sec is more likely to be in a state where it is not capable to cleave the APP fragment).

The hydration properties of PS-1 have been elucidated by counting the water molecules present at specific locations, leading to the conclusion that there is an ample amount of water molecules present in and around the active site of PS-1. The high number of polar solvent molecules in the putative binding site also hinted at the possible destabilization of a hydrophobic substrate helix, not only in the location of the scissile bond but also further downstream up to the substrate C-terminus.

The prime suspect to be the main substrate binding site is the cavity located between TMDs 3 and 5. Not only has it been stable throughout all simulations, it was large enough to bind a substrate TMD (Bai et al., 2015b), outlined by several FAD mutation sites (cf. www.alzforum.org) and strongly immobilized hydrophobic chains as shown by the lipid mobility computation for system 2. If C99 or other substrates bind in this groove, it would also be very likely that they formed additional interactions with the nicastrin loop extending from residue S241 to C248. What speaks against TMDs 2 and 3 forming the portal to the active site is the presence of a very short loop connecting both helices at the intracellular side, thus forcing a potential (uncleaved) substrate molecule to be kinked in order to fit. On the other hand, the gathered simulation data strongly suggests, that the protein is very flexible in exactly this region. Such high plasticity is of course very beneficial for the hypothetical entry of a sterically demanding substrate molecule. Future simulation studies in the presence of a substrate TM helix may help to elucidate putative substrate binding sites as well as entry pathways to the active site and also provide insight into how the flexibility of γ-secretase is affected by substrate binding.

## Author Contributions

MH performed research, analyzed data, and wrote the article. MZ designed research and wrote the article.

### Conflict of Interest Statement

The authors declare that the research was conducted in the absence of any commercial or financial relationships that could be construed as a potential conflict of interest.
