# Supplementary material for: γ-Secretase Studied by Atomistic Molecular Dynamics Simulations: Global Dynamics, Enzyme Activation, Water Distribution and Lipid Binding
Source: Front Chem. 2019 Jan 4;6:640. doi: 10.3389/fchem.2018.00640 (PMC6328467; doi:10.3389/fchem.2018.00640)
Supplement: Supplementary file 8 [file Data_Sheet_1.pdf]

Supporting Information: γ-Secretase Studied by Atomistic Molecular Dynamics Simulations: Global Dynamics, Enzyme Activation, Water Distribution and Lipid Binding

M. Hitzenberger and M. Zacharias*

Physics Department T38

Technical University of Munich

James-Frank-Str. 1

85748 Garching, Germany

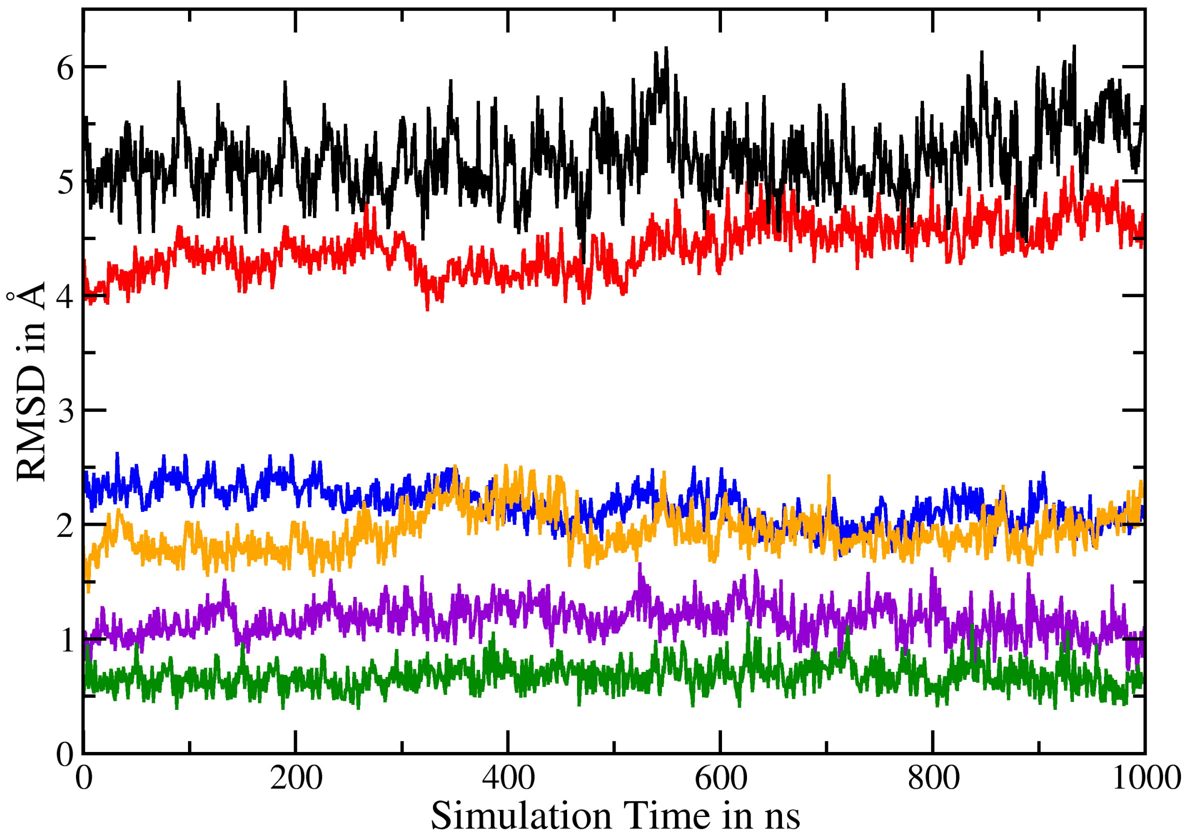

Figure 1: Cα RMSD of system 1 with PDB:5FN2 as the reference structure. Red: Complete nicastrin; blue: PS-1 TMDs; green: nicastrin TMD; purple: Pen-2 TMDs; orange: APH-1a TMDs and black: complete protein.

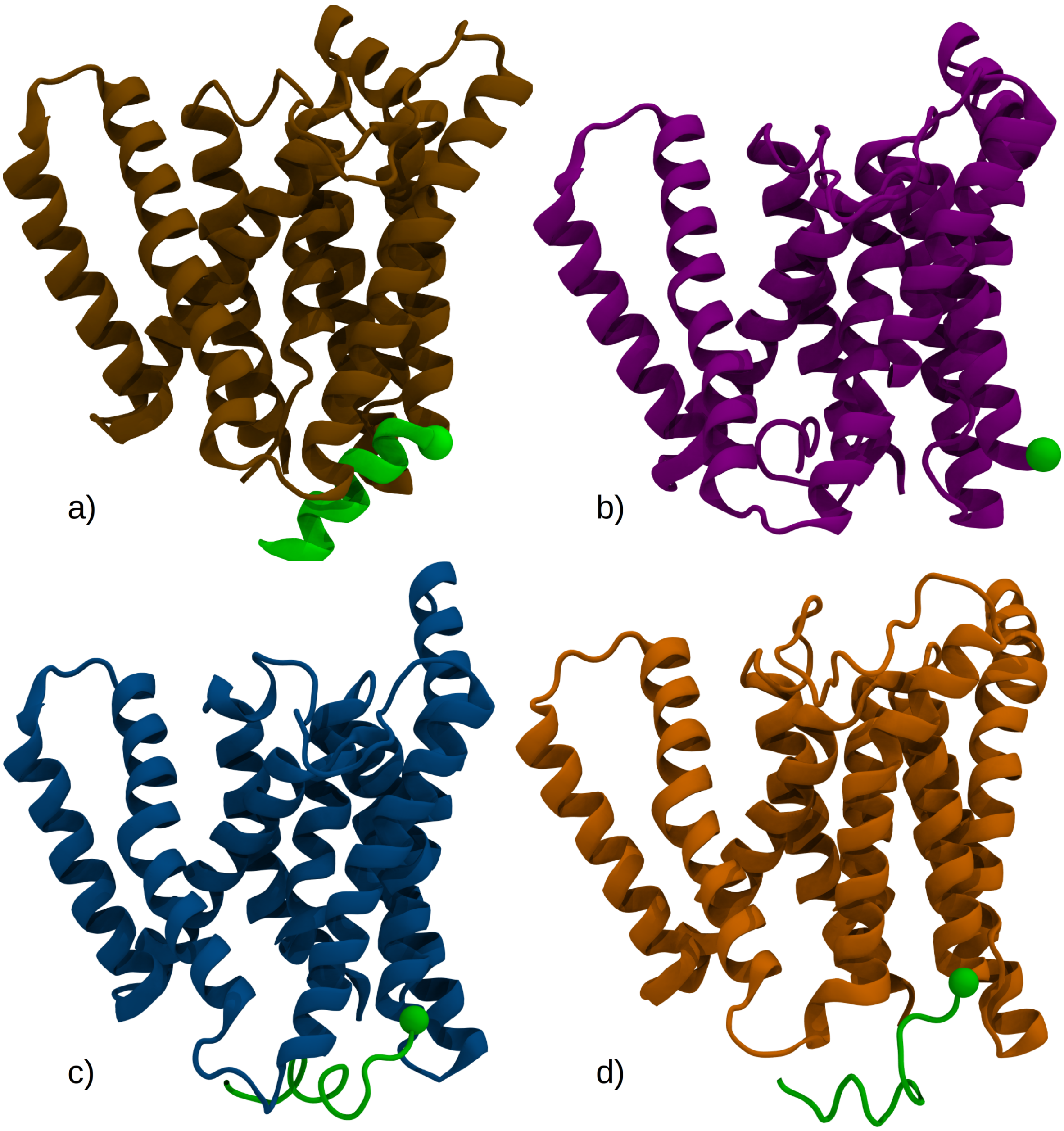

Figure 2: Comparison of Presenilin structures. C263 is marked by a green sphere. a): In the DAPT bound structure (PDB:5FN2), TMD6 remains helical beyond residue C263 (green region). b): In the PDB structure 5FN3 (Apo-state), residues C-terminal of C263 are not resolved. c): System 1, simulation started from PDB:5FN2 but after removing ligand and inhibitor coordinates, the helicity ot TMD 6 is lost beyond residue C263 (green loop-like region) during the simulation (snapshot after approximately 100 ns). d): The simulation of system 2 shows the same destabilization of the terminal part of TMD 6 during the simulation.

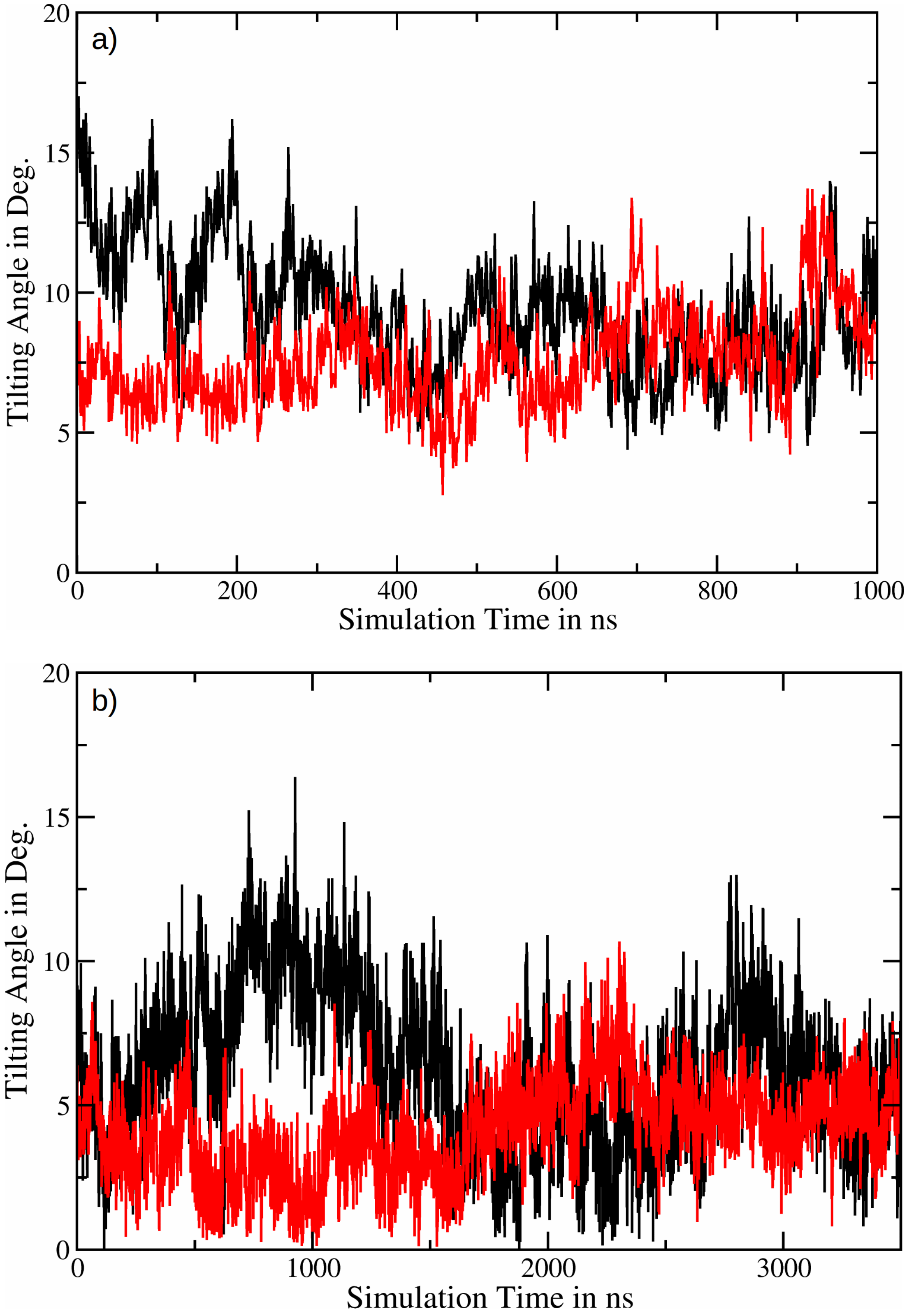

Figure 3: Tilting angles of PEN-2 (black) and Aph-1a (red). The simulation trajectories have been aligned on TMDs 4, 5, 7, 8 and 9 with respect to the first frame of each simulation. Please note that the two trajectories have not been aligned to each other, therefore the absolute values shown on both plots are not directly comparable.
a) and b) depict tilting angles of systems 1 and 2, respectively

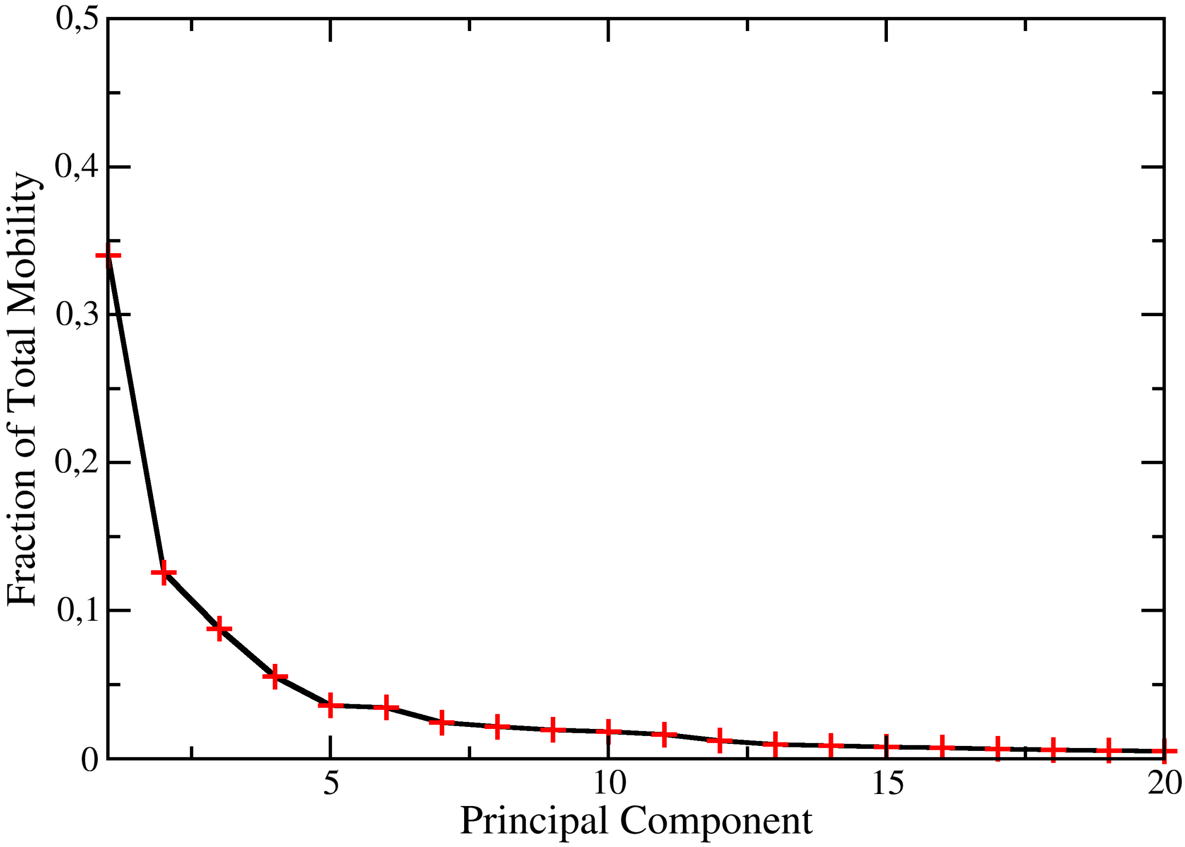

Figure 4: Fraction of total mobility in the simulation of system 1 vs. principal component number.

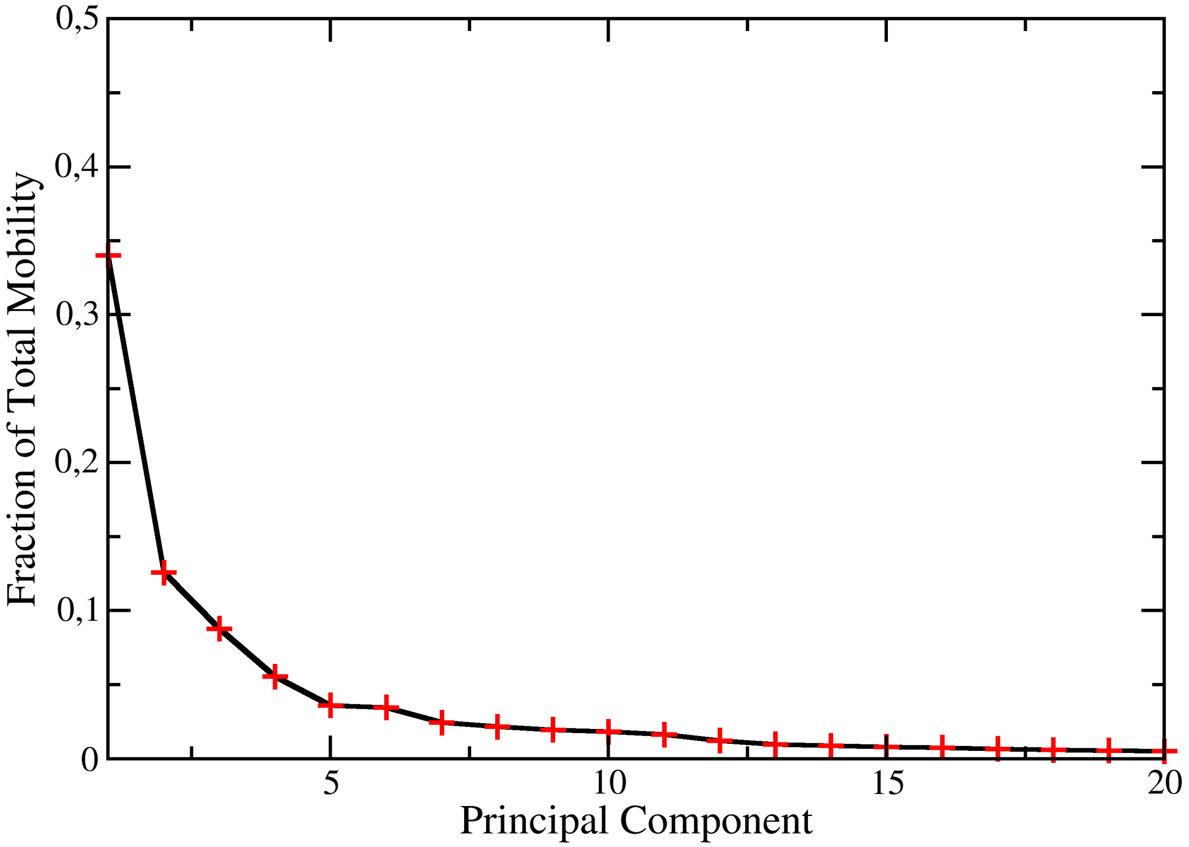

Figure 5: Fraction of total mobility in the simulation of system 2 vs. principal component number.
